# Phyllomeroterpenoids A-C, Multi-biosynthetic Pathway Derived Meroterpenoids from the TCM Endophytic Fungus *Phyllosticta* sp. and their Antimicrobial Activities

**DOI:** 10.1038/s41598-017-13407-y

**Published:** 2017-10-10

**Authors:** Heng-Gang Yang, Huan Zhao, Jiao-Jiao Li, Shao-Meng Chen, Lang-Ming Mou, Jian Zou, Guo-Dong Chen, Sheng-Ying Qin, Chuan-Xi Wang, Dan Hu, Xin-Sheng Yao, Hao Gao

**Affiliations:** 1 Institute of Traditional Chinese Medicine & Natural Products, College of Pharmacy/Guangdong Province Key Laboratory of Pharmacodynamic Constituents of TCM and New Drugs Research, Jinan University, Guangzhou, 510632 People’s Republic of China; 2 State Key Laboratory of Pharmaceutical Biotechnology, Nanjing University, Nanjing, 210023 People’s Republic of China; 3 Clinical Experimental Center, First Affiliated Hospital of Jinan University, Guangzhou, 510632 People’s Republic of China

## Abstract

Phyllomeroterpenoids A−C (**1**−**3**), multi-biosynthetic pathway derived meroterpenoids from amino acid/pentose phosphate/terpenoid pathways, were isolated from the TCM endophytic fungus *Phyllosticta* sp. J13-2-12Y, together with six biosynthetically related compounds (**4**−**9**). All structures were determined by extensive spectroscopic analysis, chemical derivatization, and ECD experiments. A plausible biosynthetic pathway of **1**−**3** was proposed. In addition, the antimicrobial activities of all isolated compounds were evaluated against *Staphylococcus aureus* 209P (bacterium) and *Candida albicans* FIM709 (fungus).

## Introduction

Meroterpenoids, such as fumagillin^1^, mycophenolic acid^2^, avinosol^3^, merochlorin A^4^, cochlearol B^5^, and others, have received much attention from chemists and pharmacologists^6^ for their remarkable structural diversity and varied biological activities. In general, meroterpenoids originate from a dual-biosynthetic pathway^6,7^. This is composed of a non-terpenoid pathway and a terpenoid pathway, such as the polyketide/terpenoid and shikimate/terpenoid pathways.

**Figure 1:**
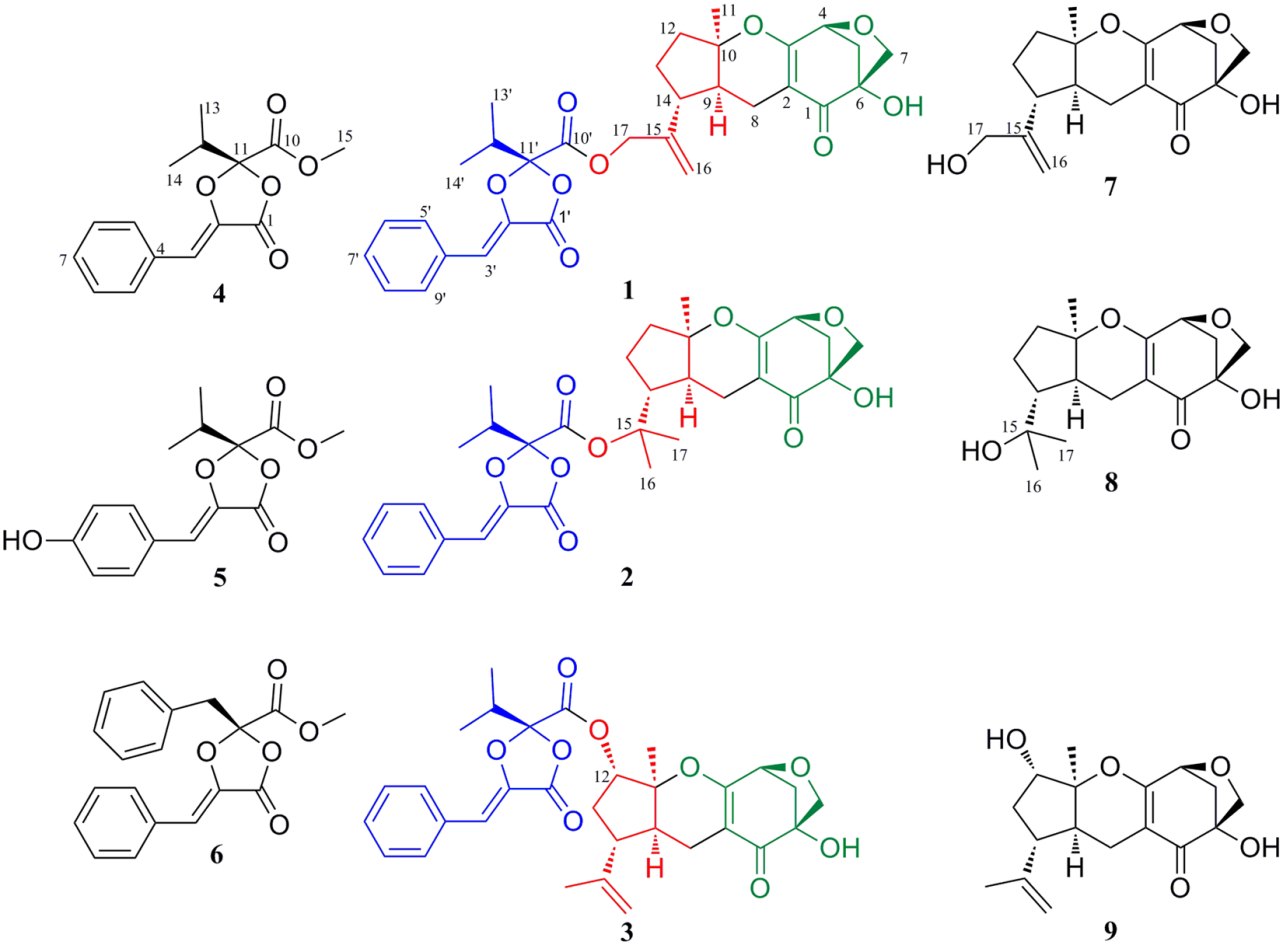
Chemical structures of **1**−**9**.

*Acorus tatarinowii* is a common and important medicinal plant, and its dried rhizomes have a long history of being used as traditional Chinese medicine (TCM) as Shi Chang Pu (Acori Tatarinowii Rhizoma) to treat many diseases, such as nervous ailments, dysentery, bronchitis, intermittent fevers ect^8^. *A. tatarinowii* is rich with asarones, which show antimicrobial activity^8,9^. The micro-environment of *A. tatarinowii* is special due to the existence of abundant antimicrobial asarones, and the microorganisms living in this habitat should be distinctive. During our recent search for bioactive compounds from microorganisms^10–14^, chemical investigation on a TCM endophytic fungal strain of *Phyllosticta* sp. J13-2-12Y from the leaves of *A. tatarinowii* was carried out. Through this investigation, three unusual meroterpenoids, phyllomeroterpenoids A–C (**1**−**3**), were isolated, along with six biosynthetically related compounds (**4**−**9**) (Fig. 1). Phyllomeroterpenoids A–C (**1**−**3**) are multi-biosynthetic pathway derived meroterpenoids, whose structures are composed of one guignardianone unit from the amino acid pathway, one C7 unit from the pentose phosphate pathway, and one monoterpene unit from the terpenoid pathway. The guignardianone unit is the skeleton of the guignardianone derivative, while the C7 unit and the monoterpene unit compose the guignardone-type meroterpenoids. In this study, we report the isolation and structural elucidation of **1**−**9** as well as their antimicrobial activities. In addition, a plausible biogenetic pathway of **1**−**3** is proposed.

## Results

The known compounds, including three guignardianones (**4**−**6**) and three guignardone-type meroterpenoids (**7**−**9**) were identified as (*S*, *Z*)-guignardianone C (**4**)^15,16^, (*S*, *Z*)-botryosphaerin B (**5**)^17^, (*S*, *Z*)-phenguignardic acid methyl ester (**6**)^15,16^, (4*S*, 6*R*, 9*S*, 10*R*, 14*R*)−17-hydroxylated guignardone A (**7**)^18^, (4*S*, 6*R*, 9*S*, 10*R*, 14*R*)-guignardone B (**8**)^19^, and (4*S*, 6*R*, 9*S*, 10*S*, 12*S*, 14*R*)−12-hydroxylated guignardone A (**9**)^18^ by comparisons of their NMR (recorded in CDCl_3_) and ECD data with references. In addition, the NMR data of **4** in CD_3_OD (Tables S4), **8** in CD_3_OD (Tables S5), and **9** in DMSO-*d*_6_ (Tables S6) are reported for the first time.

**Figure 2:**
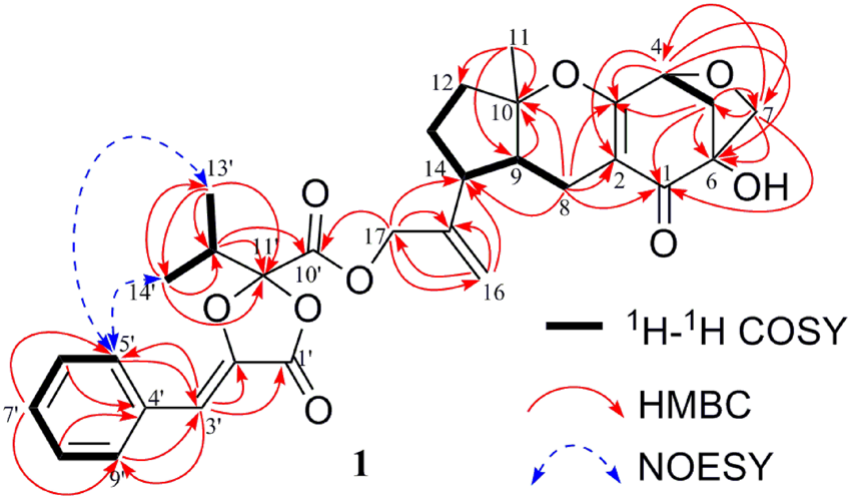
Key^1^H-^1^H COSY, HMBC, and NOESY correlations of **1**.

Phyllomeroterpenoid A (**1**) was obtained as a yellowish oil. The positive ion at *m/z* 551.2277 [M + H]^+^ (calcd. for C_31_H_35_O_9_, 551.2281) from HRESIMS indicated the molecular formula of C_31_H_34_O_9_ (index of hydrogen deficiency = 15). In the^1^H NMR spectrum of **1**, the characteristic signals of five aromatic protons [*δ*_H_ 7.67 (2H), 7.41 (2H), 7.35 (1H)], three olefinic protons [*δ*_H_ 6.50 (1H, s), 5.11 (1H, br s), 4.91 (1H, br s)], one *O*-methine [*δ*_H_ 4.54 (1H, d, *J* = 5.5 Hz)], two *O*-methylenes [*δ*_H_ 4.64 (2H, br s), 3.79 (1H, d, *J* = 7.9 Hz), 3.47 (1H, d, *J* = 7.9 Hz)], and three methyls [*δ*_H_ 1.29 (3H, s), 1.07 (6H, d, *J* = 6.9 Hz)] were observed. Among them, the five aromatic protons indicated the existence of a mono-substituted benzene ring moiety in **1**. Combined with the DEPT-135 spectrum, 31 signals were observed in the^13^C NMR spectrum, which can be assigned to eight sp^2^ quaternary carbons (including one ketone carbonyl and two ester carbons), six sp^2^ methine carbons, one sp^2^ methylene carbon, three sp^3^ *O*-quaternary carbons, four sp^3^ methine carbons (including one *O*-methine carbon), six sp^3^ methylene carbons (including two *O*-methylene carbons), and three methyl carbons. Based on the analysis of^1^H−^1^H COSY experiment, four subunits (C-4—C-5, C-8—C-9—C-14—C-13—C-12, C-5′—C-6′—C-7′—C-8′—C-9′, and C-13′—C-12′—C-14′) were revealed as shown in Fig. 2. Combined with the analysis of^1^H−^1^H COSY, the HMBC correlations (Fig. 2) from H-4 to C-2/C-3/C-6/C-7, from Ha-5/Hb-5 to C-1/C-3/C-6/C-7, from Ha-7/Hb-7 to C-1/C-4/C-5/C-6, from Ha-8/Hb-8 to C-1/C-2/C-3/C-10/C-14, from H-9 to C-10, from H_3_-11 to C-9/C-10/C-12, from Ha-16/Hb-16 to C-14/C-15/C-17, and from H_2_-17 to C-14/C-15/C-16 revealed a guignardone-type meroterpenoid moiety in **1**. In addition, the HMBC correlations (Fig. 2) from H-3′ to C-1′/C-2′/C-5′/C-9′, from H-5′/H-9′ to C-3′, from H-6′/H-8′ to C-4′, from H-7′ to C-5′/C-9′, from H-12′ to C-10′/C-11′, from H_3_-13′ to C-11′/C-12′/C-14′, and from H_3_-14′ to C-11′/C-12′/C-13′ revealed a guignardianone moiety in **1**, combined with a comparison of NMR data with (*S, Z*)-guignardianone C (**4**)^15,16^ and the above analysis of^1^H−^1^H COSY. Based on the molecular formula and the key HMBC correlation from H_2_-17 to C-10′, these two moieties can be combined, and the planar structure was established as shown in Fig. 2. This is the ester of a guignardone-type meroterpenoid and a guignardianone, and the assignments of NMR data can be found in Table 1.

**Table 1:** NMR data of 1−3 (*δ* in ppm, *J* in Hz).

No.	1^a^		2^a^		3^a^	
	*δ*_C_, mult	^c^ *δ*_H_ (*J* in Hz)	*δ*_C_, mult	^c^ *δ*_H_ (*J* in Hz)	*δ*_C_, mult	^c^ *δ*_H_ (*J* in Hz)
1	198.6, C		200.1, C		198.7, C	
2	102.7, C		104.4, C		104.0, C	
3	172.7, C		174.3, C		171.8, C	
4	78.3, CH	4.54, d (5.5)	79.9, CH	4.52, d (5.5)	78.0, CH	4.53, d (5.5)
5	44.0, CH_2_	2.45, dd (10.7, 5.5), Ha	45.1, CH_2_	2.30, dd (10.7, 5.5), Ha	43.9, CH_2_	2.45, Ha
		2.02, d (10.7), Hb		2.07, d (10.7), Hb		2.00, d (10.7), Hb
6	81.7, C		83.3, C		81.9, C	
7	70.5, CH_2_	3.79, d (7.9), Ha	72.3, CH_2_	3.70, d (7.9), Ha	70.5, CH_2_	3.80, d (7.9), Ha
		3.47, d (7.9), Hb		3.49, d (7.9), Hb		3.45, d (7.9), Hb
8	15.5, CH_2_	2.33, br d (17.1), Ha	18.7, CH_2_	2.55, dd (17.1, 1.3), Ha	15.0, CH_2_	2.29, Ha
		2.21, Hb		2.21, dd (17.1, 5.9), Hb		2.04, Hb
9	43.5, CH	1.99	42.3, CH	2.11, ddd (10.0, 5.9, 1.3)	40.7, CH	2.06
10	88.8, C		91.7, C		88.7, C	
11	23.0, CH_3_	1.29, s	22.9, CH_3_	1.28, s	18.4, CH_3_	1.14, s
12	37.0, CH_2_	2.07, ddd (14.1, 9.2, 4.0), Ha	38.6, CH_2_	1.89, ddd (13.1, 7.7, 1.0), Ha	80.8, CH	5.23, dd (6.8, 2.1)
		1.77, ddd (14.1, 11.7, 6.2), Hb		1.67, Hb		
13	27.4, CH_2_	1.99, Ha	25.3, CH_2_	1.79, Ha	35.2, CH_2_	2.54, ddd (15.3, 9.8, 6.9), Ha
		1.51, Hb		1.55, Hb		1.51, ddd (15.3, 8.1, 2.2), Hb
14	45.3, CH	2.21	51.1, CH	1.93, td (10.0, 4.8)	47.1, CH	2.16
15	142.7, C		90.3, C		143.3, C	
16	114.9, CH_2_	5.11, br s, Ha	24.3,^*2^ CH_3_	1.51,^*3^ s	113.0, CH_2_	4.75, br s, Ha
		4.91, br s, Hb				4.59, br s, Hb
17	67.4, CH_2_	4.64, br s	24.2,^*2^ CH_3_	1.50,^*3^ s	18.5, CH_3_	1.60, br s
1′	162.7, C		164.2, C		162.7, C	
2′	135.6, C		137.3, C		135.6, C	
3′	109.8, CH	6.50, s	110.2, CH	6.52, s	110.0, CH	6.53, s
4′	132.1, C		133.7, C		132.0, C	
5′/9′	129.9, CH	7.67	130.9, CH	7.70	129.8, CH	7.65
6′/8′	128.8, CH	7.41	129.9, CH	7.41	128.9, CH	7.40
7′	129.1, CH	7.35	130.3, CH	7.35	129.4, CH	7.36
10′	165.0, C		165.6, C		164.2, C	
11′	108.4, C		109.9, C		108.3, C	
12′	32.9, CH	2.67, sept (6.9)	33.8, CH	2.61, sept (6.9)	32.5, CH	2.71, sept (6.9)
13′*^1^	15.2, CH_3_	1.07, d (6.9)	15.6, CH_3_	1.06, d (6.9)	15.2, CH_3_	1.11, d (6.9)
14′*^1^	14.5, CH_3_	1.07, d (6.9)	14.8, CH_3_	1.04, d (6.9)	14.6, CH_3_	1.11, d (6.9)

^a^The data recorded in CDCl_3_ (600 MHz for ^1^H and 150 MHz for ^13^C).

^b^The data recorded in CD_3_OD (600 MHz for ^1^H and 150 MHz for ^13^C).

^c^Indiscernible signals from overlap or complex multiplicity are reported without designating multiplicity.

^*^The assignment maybe exchanged in each group.

The key NOESY correlations (Table S1) between H-5′/H-9′ and H_3_-13′/H_3_-14′ indicated that the configuration of the double bond of ∆^2′^ as *Z*. Furthermore, the^13^C NMR data of guignardianone unit in **1** were quite similar to those of (*S, Z*)-guignardianone C (**4**)^14,16^, which confirmed the above deduction. In addition, the alkaline hydrolysis of **1** give a major reaction product (**1a**) that was identified as (4*S*, 6*R*, 9*S*, 10*R*, 14*R*)−17-hydroxylated guignardone A (**7**)^18^ by HPLC, NMR data, and electronic circular dichroism (ECD) comparisons (Figures S1–S3). Thus, the absolute configurations of C-4, C-6, C-9, C-10, and C-14 in **1** were deduced to be the same as those in **7**.

**Figure 3:**
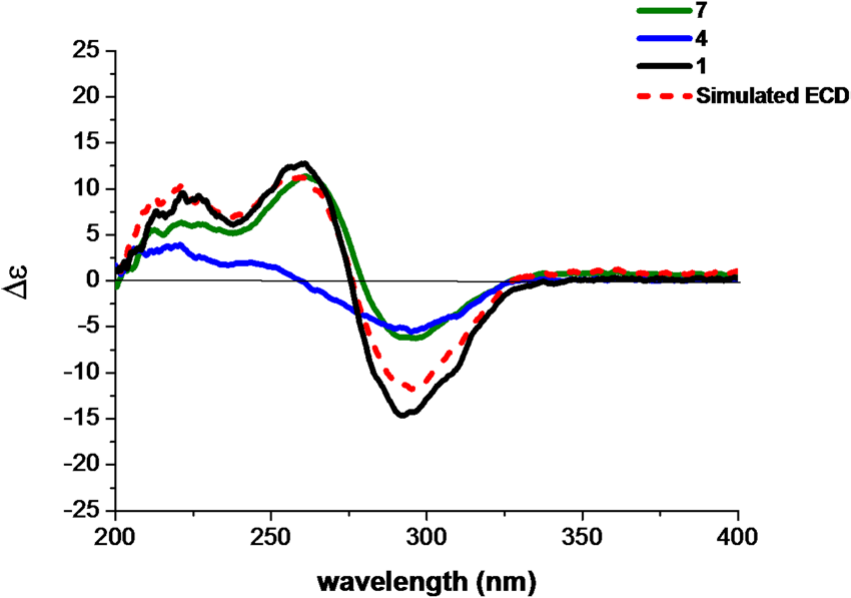
The experimental ECD spectra of **1**, **4**, and **7** and the simulated ECD spectrum of **1** (the sum of **4** and **7**).

The structure of **1** is composed of a guignardone moiety and a guignardianone moiety, so the observed ECD of **1** should result from the summed contributions of these two moieties based on the ECD additivity rule in diketones^19^. According to the structure, **7** can represent the contribution of the guignardone moiety, while **4** or the enantiomer of **4** can represent the contribution of the guignardianone moiety. The simulated ECD curve of **1**, which was the sum of the experimental ECD data of **4** and **7**, was similar to that of the experimental ECD curve of **1** (Fig. 3), therefore we deduced that the configuration of C-11′ in **1** should be the same as that in **4**. Thus, the absolute configuration of **1** was determined as 4*S*, 6*R*, 9*S*, 10*R*, 14*R*, 11′*S*.

**Figure 4:**
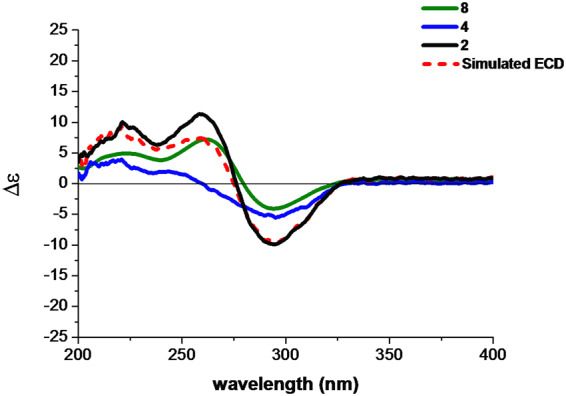
The experimental ECD spectra of **2**, **4**, and **8** and the simulated ECD spectrum of **2** (the sum of **4** and **8**).

Phyllomeroterpenoid B (**2**) was obtained as a yellowish oil. The positive ion at *m/z* 553.2455 [M + H]^+^ (calcd. for C_31_H_37_O_9_, 553.2438) from HRESIMS indicated the molecular formula of C_31_H_36_O_9_ (index of hydrogen deficiency = 14). The detailed 2D NMR analysis (Table S2) and the comparison of NMR data with (*S*, *Z*)-guignardianone C (**4**) (Table S4) revealed that **2** was the ester of a guignardone-type meroterpenoid and a guignardianone unit. A precise comparison of 1D NMR data of **2** (Table 1) with (4*S*, 6*R*, 9*S*, 10*R*, 14*R*)-guignardone B (**8**) (Table S5) showed an obviously downfield shifted carbon at C-15, which suggested that the esterification was at C-15 in **2**. Therefore, the planar structure of **2** was established as shown in Fig. 1. Combined with the carbon NMR data comparison with (*S*, *Z*)-guignardianone C (**4**) (Table S4), the key NOESY correlations (Table S2) between H-5′/H-9′ and H_3_-13′/H_3_-14′ revealed the configuration of the double bond of ∆^2′^ as *Z*. With the same alkaline hydrolysis experiment (Figures S4–S6) and the comparison analysis of the simulated ECD with the experimental ECD data (Fig. 4) as described in **1**, the absolute configuration of **2** was determined as 4*S*, 6*R*, 9*S*, 10*R*, 14*R*, 11′*S*.

Phyllomeroterpenoid C (**3**) was obtained as a yellowish oil, and its molecular formula was the same as that of **1** (C_31_H_34_O_9_) as determined by HRESIMS. Based on comparison of the NMR data with (*S*, *Z*)-guignardianone C (**4**)^15,16^ and detailed NMR analyses (Table S3), the planar structure of **3** was established as shown in Fig. 1, and the assignments of NMR data can be found in Table 1.

**Figure 5:**
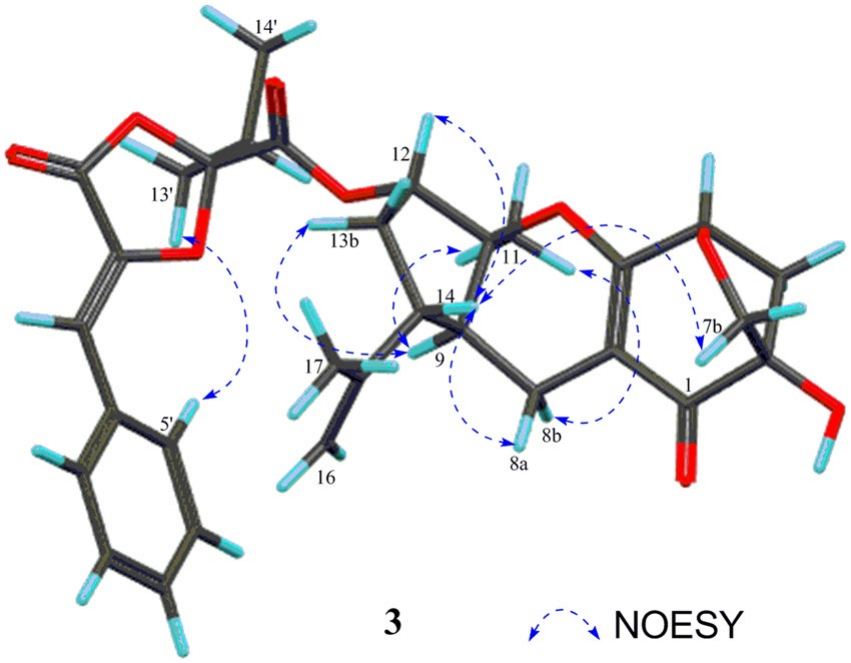
Key NOESY correlations of **3**.

Combined with the carbon NMR data comparison with (*S*, *Z*)-guignardianone C (**4**)^15,16^, the key NOESY correlations (Fig. 5) between H-5′/H-9′ and H_3_-13′/H_3_-14′ indicated the configuration of the double bond of ∆^2′^ as *Z*. In addition, the key NOESY correlations (Fig. 5) between H_3_-11 and H-9/Hb-8, between Hb-13 and H-9, between Ha-8 and H-14, between H-12 and H-14, and between H-14 and Hb-7, and the coupling constants of^3^ *J*_H-12, Hb-13_ (2.2 Hz) and^3^ *J*_H-12, Ha-13_ (6.9 Hz) in **3** were the same as those in **9** (Table S6), indicating that the relative configuration of the guignardone moiety in **3** is 4S*, 6R*, 9S*, 10S*, 12S*, and 14R*, which is the same as **9**. Since **3** and **9** coexist in *Phyllosticta* sp. J13-2-12Y, the configurations of C-4, C-6, C-9, C-10, C-12, and C-14 in **3** should be the same as those in **9**.

**Figure 6:**
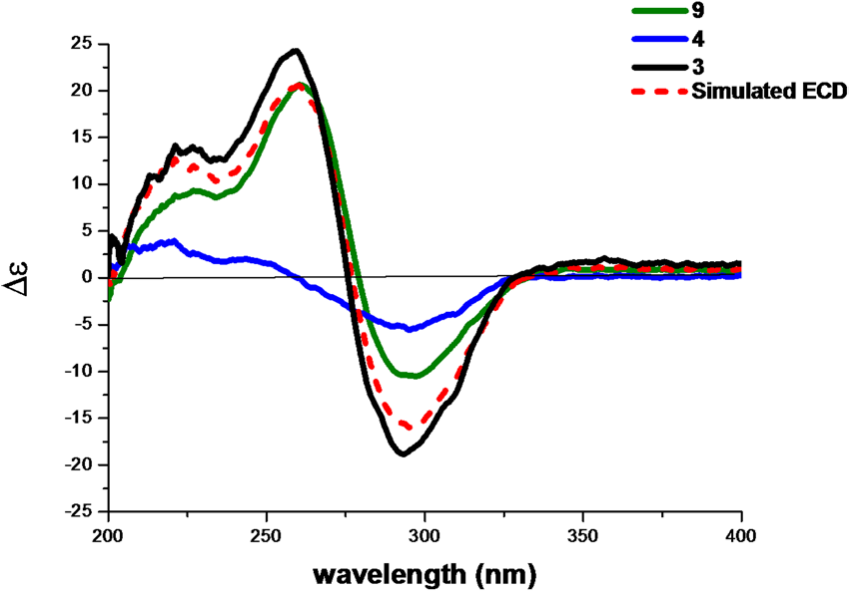
The experimental ECD spectra of **3**, **4**, and **9** and the simulated ECD spectrum of **3** (the sum of **4** and **9**).

With the same comparison analysis of the simulated ECD with the experimental ECD data (Fig. 6) as described in **1**, the absolute configuration of **3** was determined as 4*S*, 6*R*, 9*S*, 10*S*, 12*S*, 14*R*, 11′*S*.

The antimicrobial activities of the isolated compounds were evaluated against *Staphylococcus aureus* 209P (bacterium) and *Candida albicans* FIM709 (fungus). All compounds exhibited different antimicrobial activities (Table 2). Especially, **6** displayed obvious antimicrobial activities against *S. aureus* 209P and *C. albicans* FIM709 with MIC values of 4 μg/mL.

**Table 2:** The antimicrobial activities of 1–9.

Anti-*S*. *aureus* assay		Anti-*C*. *albicans* assay	
Comp.	MIC (μg/mL)	Comp.	MIC (μg/mL)
**1**	64	**1**	128
**2**	64	**2**	128
**3**	32	**3**	128
**4**	128	**4**	128
**5**	64	**5**	128
**6**	4	**6**	4
**7**	128	**7**	128
**8**	128	**8**	128
**9**	64	**9**	128
^*^Tobramycin	0.5	^*^Itraconazole	0.5

^*^Positive control.

## Discussion

**Figure 7:**
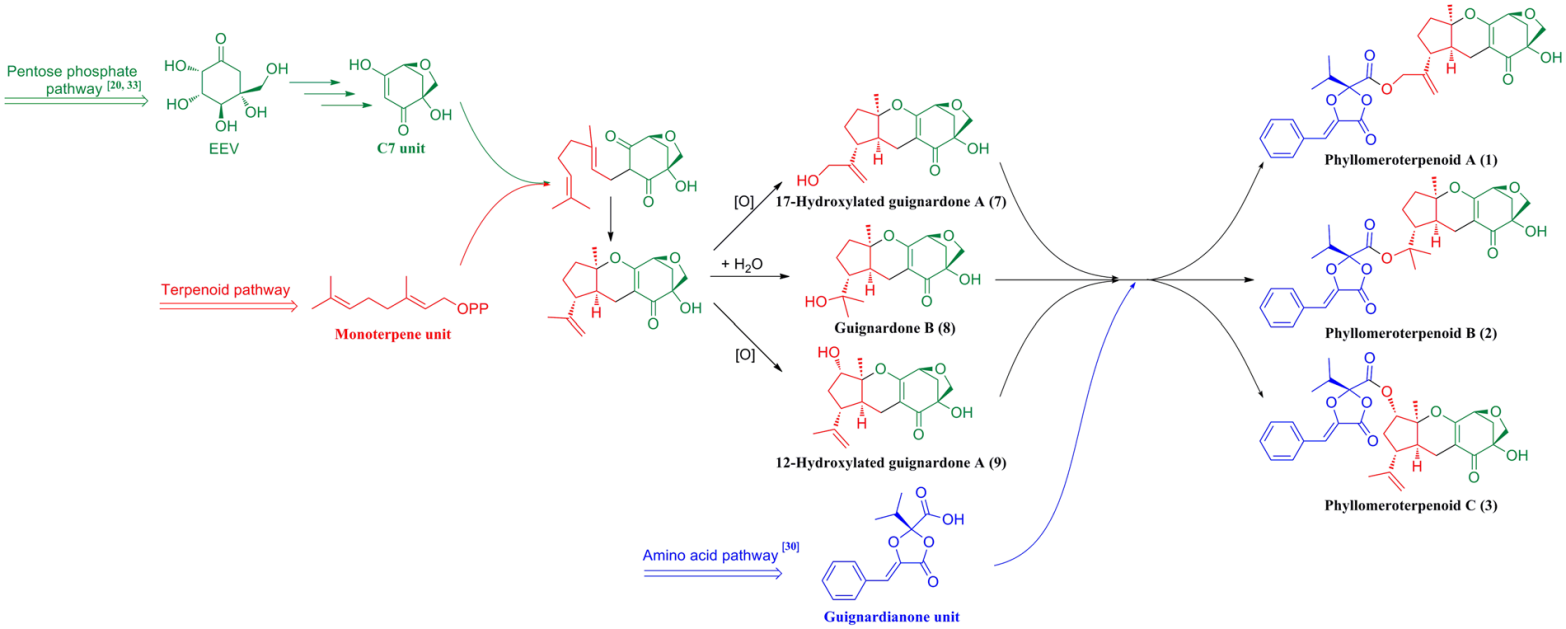
Plausible biosynthetic pathway of compounds **1**−**3**.

Guignardone-type meroterpenoids are a rare kind of meroterpenoids that are composed of one C7 unit and one monoterpene unit derived from the pentose phosphate/terpenoid pathways^20^. Up to present, about 30 members^18,21–28^ with tricyclic or tetracyclic skeletons have been reported from *Guignardia* sp.^18,21–26^, *Pycnoporus sanguineus* ^27^, and *Aspergillus* sp.^28^, and they showed antifungal^18^, antibacterial^23^, cytotoxic^25^, and Toll-Like Receptor 3 regulating activities^26^. Guignardianones are a special kind of fungal-derived benzylidene dioxolanones derived from the amino acid pathway^15,29,30^, and they exhibit antifungal^18^ and antibacterial^31^ activities. Up to now, only 13 natural guignardianones have been reported from *Guignardia* sp.^15,16,18,28,31,32^, *Botryosphaeria* sp.^17^ and *Aspergillus* sp.^30^. On the basis of our chemical investigation, three known guignardianones (**4**–**6**), and three known guignardone-type meroterpenoids (**7**–**9**) were isolated from the TCM endophytic fungal strain of *Phyllosticta* sp. J13-2-12Y. In addition, unusual meroterpenoids (**1**–**3**), the heterozygotes of guignardianone and guignardone-type meroterpenoid were also obtained. Phyllomeroterpenoids A–C (**1**–**3**) are composed of one guignardianone unit, one C7 unit, and one monoterpene unit, and they are multi-biosynthetic pathway derived meroterpenoids from the amino acid/pentose phosphate/terpenoid pathways. They could originate from phenyalanine^30^, 2-*epi*−5-*epi*-valiolone (EEV)^33^, and a monoterpenoid as shown in Fig. 7.

## Materials and Methods

### General Experimental Procedures

The detail of instruments applied in this work are provided in supporting information.

### Fungal Material

The strain numbered as J13-2-12Y was isolated from the leaves of *A. tatarinowii* collected from Guangxi Medicinal Botanical Garden, Guangxi Province, People’s Republic of China. The details of isolation and identification can be found in supporting information.

### Extraction and Isolation

The fermented material was extracted with EtOAc for three times, and the solvent was evaporated to dryness under vacuum to obtain a crude extract (42.8 g). Then the crude extract was separated by silica gel CC (4 × 15 cm) with a elution system of cyclohexane-MeOH (100:0 and 0:100, v/v) to yield a cyclohexane portion (C, 24.7 g) and a MeOH portion (W, 15.7 g). The MeOH portion (W, 15.7 g) was subjected to ODS CC (4 × 30 cm) eluting with MeOH–H_2_O (50:50, 70:30, 85:15, and 100:0, v/v) to yield 4 fractions (W1–W4). Fraction W2 (3.2 g) was further separated by MPLC on ODS CC (4 × 45 cm) with a elution system of MeOH-H_2_O (30:70 to 100:0, v/v) for 420 min at 20 mL/min to afford fractions W2-1–W2-6. Fraction W2-6 (0.4 g) was separated by silica gel CC using a elution system of cyclohexane- EtOAc (100:0 to 0:100, v/v) to yield 7 fractions (W2-6-1–W2-6-7). **1** (t_R_:66.0 min, 5.0 mg) was isolated from fraction W2-6-2 (20.0 mg) by preparative HPLC using MeCN–H_2_O (55:45, v/v) at 3 mL/min. **2** (t_R_: 58.0 min, 4.0 mg) was obtained from fraction W2-6-4 (15.0 mg) by preparative HPLC with MeCN–H_2_O (55:45, v/v) at 3 mL/min. **3** (t_R_: 38.5 min, 1.5 mg) was separated from fraction W2-6-7 (19.0 mg) by preparative HPLC using MeCN–H_2_O (60:40, v/v) at 3 mL/min. **5** (t_R_: 36.6 min, 7.0 mg) was isolated from fraction W2-2 (0.6 g) by preparative HPLC using MeOH–H_2_O (50:50, v/v) at 3 mL/min. **4** (t_R_: 11.0 min, 270.0 mg) was isolated from fraction W2–4 (0.6 g)by preparative HPLC using MeOH–H_2_O (69:31, v/v) at 3 mL/min. **6** (t_R_: 11.0 min, 85.0 mg) was isolated from fraction W2–5 (0.4 g) by preparative HPLC using MeCN–H_2_O (55:45, v/v) at 3 mL/min. Fraction W1 (4.6 g) was further separated by MPLC on ODS CC (4 × 45 cm) eluted with MeOH-H_2_O (20:80 to 100:0, v/v) for 400 min at 20 mL/min to afford 6 fractions (W1-1–W1–6). Fraction W1-2 (0.7 g) was subjected to silica gel CC with a elution system of cyclohexane-EtOAc (100:0 to 0:100, v/v) to obtain 4 fractions (W1-2-1–W1-2-4). **8** (t_R_: 18.8 min, 44.0 mg) was isolated from fraction W1-2-3 (122.0 mg) by preparative HPLC using MeCN–H_2_O (25:75, v/v) at 3 mL/min to yield. **7** (t_R_: 14.7 min, 4.0 mg) and **9** (t_R_: 15.5 min, 5.0 mg) were isolated from fraction W1-2-2 (87.0 mg) by preparative HPLC using MeCN–H_2_O (28:72, v/v) at 3 mL/min.

### Spectroscopic data of 1–3

Phyllomeroterpenoid A (**1**): yellowish oil; [*α*]^27^_D_ –38.3 (c 0.10, MeOH); UV (MeOH) λ_max_ (log *ε*) 204 (3.67), 222 (3.44), 269 (3.66), 295 (3.68), 308 (3.60); IR (KBr) v_max_ 3441, 2938, 1796, 1755, 1658, 1616, 1450, 1364, 1256, 1178, 1029, 977, 905, 758, 689 cm^−1^; ECD *λ*_nm_ (∆*ε*) (*c* 0.9 × 10^−4^ mol/L, MeOH) 222 (+9.53), 255 (+12.77), 292 (–14.64) nm; ESI-MS (positive): *m/z* 1123 [2M + Na]^+^, 573 [M + Na]^+^; HRESIMS (positive): *m/z* 551.2277 [M + H]^+^ (calcd. for C_31_H_35_O_9_, 551.2281).

Phyllomeroterpenoid B (**2**): yellowish oil; [*α*]^27^_D_ –18.7 (c 0.10, MeOH). UV (MeOH) λ_max_ (log *ε*) 204 (3.67), 222 (3.46), 267 (3.66), 295 (3.61), 309 (3.55). IR (KBr) v_max_ 3447, 2979, 2935, 1799, 1746, 1655, 1619, 1450, 1370, 1299, 1249, 1181, 1124, 1036, 977, 758, 693 cm^−1^; ECD *λ*_nm_ (∆*ε*) (*c* 0.9 × 10^−4^ mol/L, MeOH) 221 (+10.02), 258 (+11.33), 295 (–9.88) nm; ESI-MS (positive): *m/z* 575 [M + Na]^+^, 553 [M + H]^+^; HRESIMS (positive): *m/z* 553.2455 [M + H]^+^ (calcd. for C_31_H_37_O_9_, 553.2438).

Phyllomeroterpenoid C (**3**): yellowish oil; [*α*]^27^_D_ –45.0 (c 0.10, MeOH); UV (MeOH) λ_max_ (log *ε*) 204 (3.82), 222 (3.62), 263 (3.93), 294 (3.69), 308 (3.62); IR (KBr) v_max_ 3435, 2924, 1796, 1755, 1655, 1613, 1447, 1382, 1246, 1038, 891, 684 cm^−1^; ECD *λ*_nm_ (∆*ε*) (*c* 0.9 × 10^−4^ mol/L, MeOH) 221 (+14.16), 260 (+24.27), 293 (–18.84) nm; ESI-MS (positive): *m/z* 1123 [2M + Na]^+^, 573 [M + Na]^+^; HRESIMS (positive): *m/z* 551.2285 [M + H]^+^ (calcd. for C_31_H_35_O_9_, 551.2281).

### Alkaline hydrolysis of 1 and 2

A sample of **1** (1 mg) was treated with 2 N KOH (200 μL), THF (200 μL), and CH_3_OH (200 μL), and stirred at 25 °C for 4 h. After neutralizing with 10% HCOOH and extracting with EtOAc, the EtOAc layer was evaporated to dryness and dissolved in MeOH. Then, **1a** (0.4 mg) was isolated from the mixture by analytical HPLC (Phenomenex Gemini C18 column, 5 μm, 4.6 × 250 mm) with MeOH-H_2_O (69:31, v/v) at 1 mL/min, and its ^1^H NMR spectrum and ECD spectrum were identical with those of **7** (Figures S2 and S3).

A sample of **2** (1 mg) was treated with 2 N KOH (200 μL), THF (200 μL), and CH_3_OH (200 μL), and stirred at 25 °C for 4 h. After neutralizing with 10% HCOOH and extracting with EtOAc, the EtOAc layer was evaporated to dryness and dissolved in MeOH. Then, **2a** (0.4 mg) was isolated from the mixture by analytical HPLC (Phenomenex Gemini C18 column, 5 μm, 4.6 × 250 mm) with MeOH-H_2_O (67:33, v/v) at 1 mL/min, and its ^1^H NMR spectrum and ECD spectrum were identical with those of **8** (Figures S5 and S6).

### Antimicrobial Assay

The antimicrobial activities against *S. aureus* 209P and *C. albicans* FIM709 were measured in sterile 96-well plates using the broth microdilution method^34,35^, and the detail can be found in supporting information.

## Electronic supplementary material

Supporting information
