# Supplementary material for: Phyllomeroterpenoids A-C, Multi-biosynthetic Pathway Derived Meroterpenoids from the TCM Endophytic Fungus Phyllosticta sp. and their Antimicrobial Activities
Source: Sci Rep. 2017 Oct 10;7:12925. doi: 10.1038/s41598-017-13407-y (PMC5635028; doi:10.1038/s41598-017-13407-y)
Supplement: Supplementary file 1 — Supporting information [file 41598_2017_13407_MOESM1_ESM.doc]

**SUPPLEMENTARY INFORMATION**

Phyllomeroterpenoids A-C, Multi-biosynthetic Pathway Derived Meroterpenoids from the TCM Endophytic Fungus *Phyllosticta* sp. and their Antimicrobial Activities

Heng-Gang Yang,^1,+^ Huan Zhao,^1,+^ Jiao-Jiao Li,^1,+^ Shao-Meng Chen,^1^ Lang-Ming Mou,^1^ Jian Zou,^1^ Guo-Dong Chen,^1^ Sheng-Ying Qin,^3,*^ Chuan-Xi Wang,^1,*^ Dan Hu,^1^ Xin-Sheng Yao,^1^ Hao Gao^1,2,*^

^1^Institute of Traditional Chinese Medicine & Natural Products, College of Pharmacy / Guangdong Province Key Laboratory of Pharmacodynamic Constituents of TCM and New Drugs Research, Jinan University, Guangzhou 510632, People’s Republic of China

^2^State Key Laboratory of Pharmaceutical Biotechnology, Nanjing University, Nanjing 210023, People’s Republic of China

^3^Clinical Experimental Center, First Affiliated Hospital of Jinan University, Guangzhou 510632, People’s Republic of China

*Corresponding author: tghao@jnu.edu.cn (Hao Gao); qinshengying78@163.com (Sheng-Ying Qin); wcxjnu@163.com (Chuan-Xi Wang)

^+^These authors contributed equally to this work.

**Contents**

1. NMR assignments of 1–3∙∙∙∙∙∙∙∙∙∙∙∙∙∙∙∙∙∙∙∙∙∙∙∙∙∙∙∙∙∙∙∙∙∙∙∙∙∙∙∙∙∙∙∙∙∙∙∙∙∙∙∙∙∙∙∙∙∙∙∙∙∙∙∙∙∙∙∙∙∙∙∙∙∙∙∙∙∙∙∙∙∙∙∙∙∙∙∙∙3

2. NMR assignments of 4, 8, and 9∙∙∙∙∙∙∙∙∙∙∙∙∙∙∙∙∙∙∙∙∙∙∙∙∙∙∙∙∙∙∙∙∙∙∙∙∙∙∙∙∙∙∙∙∙∙∙∙∙∙∙∙∙∙∙∙∙∙∙∙∙∙∙∙∙∙∙∙∙∙∙∙∙∙∙∙∙∙6

**3. General experimental procedures ∙∙∙∙∙∙∙∙∙∙∙∙∙∙∙∙∙∙∙∙∙∙∙∙∙∙∙∙∙∙∙∙∙∙∙∙∙∙∙∙∙∙∙∙∙∙∙∙∙∙∙∙∙∙∙∙∙∙∙∙∙∙∙∙∙∙∙∙∙∙∙∙∙∙9**

**4. Fungal material ∙∙∙∙∙∙∙∙∙∙∙∙∙∙∙∙∙∙∙∙∙∙∙∙∙∙∙∙∙∙∙∙∙∙∙∙∙∙∙∙∙∙∙∙∙∙∙∙∙∙∙∙∙∙∙∙∙∙∙∙∙∙∙∙∙∙∙∙∙∙∙∙∙∙∙∙∙∙∙∙∙∙∙∙∙∙∙∙∙∙∙∙∙∙∙∙∙∙∙∙10**

5. Alkaline hydrolysis of 1 and 2 11

**6. Antimicrobial assay ∙∙∙∙∙∙∙∙∙∙∙∙∙∙∙∙∙∙∙∙∙∙∙∙∙∙∙∙∙∙∙∙∙∙∙∙∙∙∙∙∙∙∙∙∙∙∙∙∙∙∙∙∙∙∙∙∙∙∙∙∙∙∙∙∙∙∙∙∙∙∙∙∙∙∙∙∙∙∙∙∙∙∙∙∙∙∙∙∙∙∙∙∙∙∙14**

7. The 1D and 2D NMR spectra of 1−3 15

8. The 1D and 2D NMR spectra of 4, 8, and 9 24

1. NMR assignments of 1–3

**Table S1.** NMR data of **1** in CDCl_3_ (600 MHz for ^1^H and 150 MHz for ^13^C).

| **Position** | ***δ*_C_, mult** | ***δ*_H_ (*J* in Hz)***^a^* | **^1^H–^1^H COSY** | **HMBC** | **NOESY** |
| --- | --- | --- | --- | --- | --- |
| 1 | 198.6, C |  |  |  |  |
| 2 | 102.7, C |  |  |  |  |
| 3 | 172.7, C |  |  |  |  |
| 4 | 78.3, CH | 4.54, d (5.5) | 5a | 2, 3, 5, 6, 7 |  |
| 5 | 44.0, CH_2_ | 2.45, dd (10.7, 5.5), Ha | 4, 5b | 1, 3, 4, 6, 7 | 7a |
|  |  | 2.02, d (10.7), Hb | 5a | 1, 3, 4, 6, 7 |  |
| 6 | 81.7, C |  |  |  |  |
| 7 | 70.5, CH_2_ | 3.79, d (7.9), Ha | 7b | 1, 4, 5, 6 | 5a |
|  |  | 3.47, d (7.9), Hb | 7a | 1, 4, 5, 6 |  |
| 8 | 15.5, CH_2_ | 2.33, br d (17.1), Ha | 8b, 9 | 1, 2, 3, 9, 10, 14 | 16b |
|  |  | 2.21, Hb | 8a, 9 | 1, 2, 3, 9, 10, 14 |  |
| 9 | 43.5, CH | 1.99 | 8a, 8b, 14 | 2, 8, 10, 11, 12, 13, 14, 15 |  |
| 10 | 88.8, C |  |  |  |  |
| 11 | 23.0, CH_3_ | 1.29, s |  | 9, 10, 12 | 12b |
| 12 | 37.0, CH_2_ | 2.07, ddd (14.1, 9.2, 4.0), Ha | 12b, 13a, 13b | 9, 10, 13, 14 |  |
|  |  | 1.77, ddd (14.1, 11.7, 6.2), Hb | 12a, 13a, 13b | 11, 13, 14 | 11 |
| 13 | 27.4, CH_2_ | 1.99, Ha | 12a, 12b, 13b, 14 | 9, 10, 12, 14, 15 |  |
|  |  | 1.51, Hb | 12a, 12b, 13a, 14 | 9, 10, 12, 14, 15 | 17 |
| 14 | 45.3, CH | 2.21 | 9, 13a, 13b | 8, 9, 10, 12, 13, 15, 16, 17 |  |
| 15 | 142.7, C |  |  |  |  |
| 16 | 114.9, CH_2_ | 5.11, br s, Ha | 17 | 14, 15, 17 |  |
|  |  | 4.91, br s, Hb | 17 | 13, 14, 15, 17 | 8a |
| 17 | 67.4, CH_2_ | 4.64, br s | 16a, 16b | 14, 15, 16, 10' | 13b |
| 1' | 162.7, C |  |  |  |  |
| 2' | 135.6, C |  |  |  |  |
| 3' | 109.8, CH | 6.50, s |  | 1', 2', 5', 9' | 5'/9' |
| 4' | 132.1, C |  |  |  |  |
| 5'/9' | 129.9, CH | 7.67 | 6'/8', 7' | 3', 7' | 3', 13', 14' |
| 6'/8' | 128.8, CH | 7.41 | 5'/9', 7' | 4', 7' |  |
| 7' | 129.1, CH | 7.35 | 5', 6', 8', 9' | 5', 9' |  |
| 10' | 165.0, C |  |  |  |  |
| 11' | 108.4, C |  |  |  |  |
| 12' | 32.9, CH | 2.67, sept (6.9) | 13', 14' | 10', 11', 13', 14' |  |
| 13'^*^ | 15.2, CH_3_ | 1.07, d (6.9) | 12' | 11', 12', 14' | 5'/9' |
| 14'^*^ | 14.5, CH_3_ | 1.07, d (6.9) | 12' | 11', 12', 13' | 5'/9' |

*^a^* Indiscernible signals from overlap or complex multiplicity are reported without designating multiplicity.

*^*^* The assignment maybe exchanged.

**Table S2.** NMR data of **2** in CD_3_OD (600 MHz for ^1^H and 150 MHz for ^13^C).

| **Position** | ***δ*_C_, mult** | ***δ*_H_ (*J* in Hz)***^a^* | **^1^H–^1^H COSY** | **HMBC** | **NOESY** |
| --- | --- | --- | --- | --- | --- |
| 1 | 200.1, C |  |  |  |  |
| 2 | 104.4, C |  |  |  |  |
| 3 | 174.3, C |  |  |  |  |
| 4 | 79.9, CH | 4.52, d (5.5) | 5a | 2, 3, 5, 6, 7 |  |
| 5 | 45.1, CH_2_ | 2.30, dd (10.7, 5.5), Ha | 4, 5b | 1, 3, 4, 6, 7 | 7a |
|  |  | 2.07, d (10.7), Hb | 5a | 1, 3, 4, 6, 7 |  |
| 6 | 83.3, C |  |  |  |  |
| 7 | 72.3, CH_2_ | 3.70, d (7.9), Ha | 7b | 1, 4, 5, 6 | 5a |
|  |  | 3.49, d (7.9), Hb | 7a | 1, 4, 5, 6 | 14 |
| 8 | 18.7, CH_2_ | 2.55, dd (17.1, 1.3), Ha | 8b, 9 | 1, 2, 3, 9, 10, 14 | 14, 16/17 |
|  |  | 2.21, dd (17.1, 5.9), Hb | 8a, 9 | 1, 2, 3, 9, 10, 14 | 11 |
| 9 | 42.3, CH | 2.11, ddd (10.0, 5.9, 1.3) | 8a, 8b, 14 | 2, 8, 10, 11, 14, 15 | 11, 12b, 13b, 16/17 |
| 10 | 91.7, C |  |  |  |  |
| 11 | 22.9, CH_3_ | 1.28, s |  | 9, 10, 12 | 8b, 9, 12b |
| 12 | 38.6, CH_2_ | 1.89, ddd (13.1, 7.7, 1.0), Ha | 12b, 13a, 13b | 9, 10, 13, 14 |  |
|  |  | 1.67, Hb | 12a, 13a, 13b | 11, 13, 14 | 9, 11 |
| 13 | 25.3, CH_2_ | 1.79, Ha | 12a, 12b, 13b, 14 | 9, 10, 12, 14, 15 |  |
|  |  | 1.55, Hb | 12a, 12b, 13a, 14 | 9, 10, 12, 14, 15 | 9 |
| 14 | 51.1, CH | 1.93, td (10.0, 4.8) | 9, 13a, 13b | 8, 9, 10, 12, 13, 15, 16, 17 | 7b, 8a, 16/17 |
| 15 | 90.3, C |  |  |  |  |
| 16^*1^ | 24.3, CH_3_ | 1.51, s |  | 14, 15, 17 | 8a, 9, 14 |
| 17^*1^ | 24.2, CH_3_ | 1.50, s |  | 14, 15, 16 | 8a, 9, 14 |
| 1' | 164.2, C |  |  |  |  |
| 2' | 137.3, C |  |  |  |  |
| 3' | 110.2, CH | 6.52, s |  | 1', 2', 5', 9' | 5'/9' |
| 4' | 133.7, C |  |  |  |  |
| 5'/9' | 130.9, CH | 7.70 | 6'/8', 7' | 3', 7' | 3', 13', 14' |
| 6'/8' | 129.9, CH | 7.41 | 5'/9', 7' | 4', 7' |  |
| 7' | 130.3, CH | 7.35 | 5', 6', 8', 9' | 5', 9' |  |
| 10' | 165.6, C |  |  |  |  |
| 11' | 109.9, C |  |  |  |  |
| 12' | 33.8, CH | 2.61, sept (6.9) | 13', 14' | 10', 11', 13', 14' |  |
| 13'^*2^ | 15.6, CH_3_ | 1.06, d (6.9) | 12' | 11', 12', 14' | 5'/9' |
| 14'^*2^ | 14.8, CH_3_ | 1.04, d (6.9) | 12' | 11', 12', 13' | 5'/9' |

*^a^* Indiscernible signals from overlap or complex multiplicity are reported without designating multiplicity.

^*^ The assignment maybe exchanged in each group.

**Table S3.** NMR data of **3** in CDCl_3_ (600 MHz for ^1^H and 150 MHz for ^13^C).

| **Position** | ***δ*_C_, type** | ***δ*_H_ (*J* in Hz)***^a^* | **^1^H–^1^H COSY** | **HMBC** | **NOESY** |
| --- | --- | --- | --- | --- | --- |
| 1 | 198.7, C |  |  |  |  |
| 2 | 104.0, C |  |  |  |  |
| 3 | 171.8, C |  |  |  |  |
| 4 | 78.0, CH | 4.53, d (5.5) | 5a | 2, 3, 5, 6, 7 |  |
| 5 | 43.9, CH_2_ | 2.45, Ha | 4, 5b | 1, 3, 4, 6, 7 | 7a |
|  |  | 2.00, d (10.7), Hb | 5a | 1, 3, 4, 6, 7 |  |
| 6 | 81.9, C |  |  |  |  |
| 7 | 70.5, CH_2_ | 3.80, d (7.9), Ha | 7b | 1, 4, 5, 6 | 5a |
|  |  | 3.45, d (7.9), Hb | 7a | 1, 4, 5, 6 | 14 |
| 8 | 15.0, CH_2_ | 2.29, Ha | 8b, 9 | 1, 2, 3, 9, 10, 14 | 14, 16b |
|  |  | 2.04, Hb | 8a, 9 | 1, 2, 3, 9, 10, 14 | 11 |
| 9 | 40.7, CH | 2.06 | 8a, 8b, 14 | 2, 8, 10, 11, 13, 14, 15 | 11, 13b, 17 |
| 10 | 88.7, C |  |  |  |  |
| 11 | 18.4, CH_3_ | 1.14, s |  | 9, 10, 12 | 8b, 9 |
| 12 | 80.8, CH | 5.23, dd (6.8, 2.1) | 13a, 13b | 9, 10, 14, 10' | 14 |
| 13 | 35.2, CH_2_ | 2.54, ddd (15.3, 9.8, 6.9), Ha | 12, 13b, 14 | 9, 10, 14, 15 |  |
|  |  | 1.51, ddd (15.3, 8.1, 2.2), Hb | 12, 13a, 14 | 9, 10, 14, 15 | 9 |
| 14 | 47.1, CH | 2.16 | 9, 13a, 13b | 8, 9, 10, 12, 13, 15, 16, 17 | 7b, 8a, 12, 16b |
| 15 | 143.3, C |  |  |  |  |
| 16 | 113.0, CH_2_ | 4.75, br s, Ha | 17 | 14, 15, 17 |  |
|  |  | 4.59, br s, Hb | 17 | 13, 14, 15, 17 | 8a, 14 |
| 17 | 18.5, CH_3_ | 1.60, br s | 16a, 16b | 14, 15, 16 | 9 |
| 1' | 162.7, C |  |  |  |  |
| 2' | 135.6, C |  |  |  |  |
| 3' | 110.0, CH | 6.53, s |  | 1', 2', 5', 9' | 5'/9' |
| 4' | 132.0, C |  |  |  |  |
| 5'/9' | 129.8, CH | 7.65 | 6'/8', 7' | 3', 7' | 3', 13'/14' |
| 6'/8' | 128.9, CH | 7.40 | 5'/9', 7' | 4', 7' |  |
| 7' | 129.4, CH | 7.36 | 5', 6', 8', 9' | 5', 9' |  |
| 10' | 164.2, C |  |  |  |  |
| 11' | 108.3, C |  |  |  |  |
| 12' | 32.5, CH | 2.71, sept (6.9) | 13', 14' | 10', 11', 13', 14' |  |
| 13'^*^ | 15.2, CH_3_ | 1.11, d (6.9) | 12' | 11', 12', 14' | 5'/9' |
| 14'^*^ | 14.6, CH_3_ | 1.11, d (6.9) | 12' | 11', 12', 13' | 5'/9' |

*^a^* Indiscernible signals from overlap or complex multiplicity are reported without designating multiplicity.

*^*^* The assignment maybe exchanged.

**2. NMR assignments of 4, 8 and 9**

**Table S4.** NMR data of **4**

| **4***^a^* | | |  | **4***^b^* | | | |
| --- | --- | --- | --- | --- | --- | --- | --- |
| **Position** | ***δ*_C_, type** | ***δ*_H_ (*J* in Hz)***^c^* |  | ***δ*_C_, type** | ***δ*_H_ (*J* in Hz)***^c^* | **^1^H–^1^H COSY** | **HMBC** |
| 1 | 162.8, C |  |  | 163.9, C |  |  |  |
| 2 | 135.7, C |  |  | 137.0, C |  |  |  |
| 3 | 109.6, CH | 6.50, s |  | 110.3, CH | 6.51, s |  | 1, 2, 5, 9 |
| 4 | 132.2, C |  |  | 133.6, C |  |  |  |
| 5/9 | 129.9, CH | 7.68 |  | 130.9, CH | 7.70 | 6/8, 7 | 3, 7 |
| 6/8 | 128.8, CH | 7.41 |  | 129.8, CH | 7.40 | 5/9, 7 | 4, 7 |
| 7 | 129.1, CH | 7.35 |  | 130.2, CH | 7.35 | 5, 6, 8, 9 | 5, 9 |
| 10 | 166.0, C |  |  | 167.4, C |  |  |  |
| 11 | 108.5, C |  |  | 109.7, C |  |  |  |
| 12 | 32.9, CH | 2.69, sept (6.9) |  | 34.3, CH | 2.66, sept (6.9) | 13, 14 | 10, 11, 13, 14 |
| 13^*^ | 15.2, CH_3_ | 1.07, d (6.9) |  | 15.5, CH_3_ | 1.05, d (6.9) | 12 | 11, 12, 14 |
| 14^*^ | 14.5, CH_3_ | 1.07, d (6.9) |  | 14.8, CH_3_ | 1.04, d (6.9) | 12 | 11, 12, 13 |
| 15 | 53.4, CH_3_ | 3.85, s |  | 54.0, CH_3_ | 3.85, s |  | 10 |

*^a^* Measured in CDCl_3_ (300 MHz for ^1^H and 75 MHz for ^13^C).

*^b^* Measured in CD_3_OD (600 MHz for ^1^H and 150 MHz for ^13^C).

*^c^* Indiscernible signals from overlap or complex multiplicity are reported without designating multiplicity.

*^*^* The assignment maybe exchanged.

**Table S5.** NMR data of **8**

| **8***^a^* | | |  | **8***^b^* | | | |
| --- | --- | --- | --- | --- | --- | --- | --- |
| **Position** | ***δ*_C_, type** | ***δ*_H_ (*J* in Hz)***^c^* |  | ***δ*_C_, type** | ***δ*_H_ (*J* in Hz)***^c^* | **^1^H–^1^H COSY** | **HMBC** |
| 1 | 198.7, C |  |  | 200.4, C |  |  |  |
| 2 | 102.6, C |  |  | 104.7, C |  |  |  |
| 3 | 174.0, C |  |  | 174.6, C |  |  |  |
| 4 | 78.4, CH | 4.56, d (5.4) |  | 80.0, CH | 4.54, d (5.4) | 5a | 2, 3, 5, 6, 7 |
| 5 | 43.9, CH_2_ | 2.46, dd (10.8, 5.4), Ha |  | 45.3, CH_2_ | 2.31, dd (10.8, 5.4), Ha | 4, 5b | 1, 3, 4, 6, 7 |
|  |  | 2.03, d (10.8), Hb |  |  | 2.08, Hb | 5a | 1, 3, 4, 6, 7 |
| 6 | 81.7, C |  |  | 83.3, C |  |  |  |
| 7 | 70.6, CH_2_ | 3.81, d (7.8), Ha |  | 72.3, CH_2_ | 3.72, d (7.8), Ha | 7b | 1, 4, 5, 6 |
|  |  | 3.51, d (7.8), Hb |  |  | 3.52, d (7.8), Hb | 7a | 1, 4, 5, 6 |
| 8 | 18.2, CH_2_ | 2.63, br d (17.0), Ha |  | 19.1, CH_2_ | 2.62, br d (17.0), Ha | 8b, 9 | 1, 2, 3, 9, 10, 14 |
|  |  | 2.28, dd (17.0, 6.1), Hb |  |  | 2.22, dd (17.0, 6.1), Hb | 8a, 9 | 1, 2, 3, 9, 10, 14 |
| 9 | 41.3, CH | 2.08 |  | 42.5, CH | 2.10 | 8a, 8b, 14 | 2, 8, 10, 11, 14, 15 |
| 10 | 90.7, C |  |  | 91.8, C |  |  |  |
| 11 | 22.9, CH_3_ | 1.32, s |  | 23.0, CH_3_ | 1.33, s |  | 9, 10, 12 |
| 12 | 38.4, CH_2_ | 2.00, Ha |  | 39.3, CH_2_ | 1.95, Ha | 12b, 13a, 13b | 9, 10, 13, 14 |
|  |  | 1.64, Hb |  |  | 1.68, Hb | 12a, 13a, 13b | 11, 13, 14 |
| 13 | 24.6, CH_2_ | 1.86, Ha |  | 25.5, CH_2_ | 1.78, Ha | 12a, 12b, 13b, 14 | 9, 10, 12, 14, 15 |
|  |  | 1.54, Hb |  |  | 1.61, Hb | 12a, 12b, 13a, 14 | 9, 10, 12, 14, 15 |
| 14 | 51.0, CH | 1.56 |  | 52.5, CH | 1.55 | 9, 13a, 13b | 8, 9, 10, 12, 13, 15, 16, 17 |
| 15 | 72.8, C |  |  | 73.5, C |  |  |  |
| 16^*^ | 28.7, CH_3_ | 1.21, s |  | 28.3, CH_3_ | 1.16, s |  | 14, 15, 17 |
| 17^*^ | 27.5, CH_3_ | 1.18, s |  | 27.1, CH_3_ | 1.14, s |  | 14, 15, 16 |

*^a^* Measured in CDCl_3_ (300 MHz for ^1^H and 75 MHz for ^13^C).

*^b^* Measured in CD_3_OD (400 MHz for ^1^H and 100 MHz for ^13^C).

*^c^* Indiscernible signals from overlap or complex multiplicity are reported without designating multiplicity.

*^*^* The assignment maybe exchanged.

**Table S6.** NMR data of **9.**

|  | **9***^a^* | |  | **9***^b^* | | | | |
| --- | --- | --- | --- | --- | --- | --- | --- | --- |
| **Position** | ***δ*_C_, type** | ***δ*_H_ (*J* in Hz)*^c^*** |  | ***δ*_C_, type** | ***δ*_H_ (*J* in Hz)*^c^*** | **^1^H–^1^H COSY** | **HMBC** | **ROESY** |
| 1 | 198.8, C |  |  | 198.2, C |  |  |  |  |
| 2 | 103.9, C |  |  | 104.2, C |  |  |  |  |
| 3 | 172.5, C |  |  | 170.5, C |  |  |  |  |
| 4 | 78.3, CH | 4.54, d (5.4) |  | 77.7, CH | 4.49, d (5.4) | 5a | 2, 3, 5, 6, 7 |  |
| 5 | 44.0, CH_2_ | 2.47, Ha |  | 44.0, CH_2_ | 2.16, dd (10.7, 5.4), Ha | 4, 5b | 1, 3, 4, 6, 7 | 7a |
|  |  | 2.04, d (10.8), Hb |  |  | 2.06, Hb | 5a | 1, 3, 4, 6, 7 |  |
| 6 | 81.8, C |  |  | 81.9, C |  |  |  |  |
| 7 | 70.5, CH_2_ | 3.81, d (7.8), Ha |  | 71.1, CH_2_ | 3.59, d (7.8), Ha | 7b | 1, 4, 5, 6 | 5a |
|  |  | 3.48, d (7.8), Hb |  |  | 3.38, d (7.8), Hb | 7a | 1, 4, 5, 6 | 14 |
| 8 | 15.3, CH_2_ | 2.34, br d (16.1), Ha |  | 15.1, CH_2_ | 2.06, Ha | 8b, 9 | 1, 2, 3, 9, 10, 14 | 14, 16b |
|  |  | 2.15, Hb |  |  | 2.02, dd (17.1, 6.1), Hb | 8a, 9 | 1, 2, 3, 9, 10, 14 | 11 |
| 9 | 39.9, CH | 2.19 |  | 39.5, CH | 2.09 | 8a, 8b, 14 | 2, 8, 10, 11, 13, 14, 15 | 11, 12-OH, 13b, 17 |
| 10 | 90.2, C |  |  | 89.9, C |  |  |  |  |
| 11 | 18.9, CH_3_ | 1.32, s |  | 18.7, CH_3_ | 1.21, s |  | 9, 10, 12 | 8b, 9, 12-OH |
| 12 | 77.2, CH | 4,14, dd (6.8, 2.3) |  | 75.7, CH | 3.91, ddd (6.8, 4.7, 2.6) | 12-OH, 13a, 13b | 9, 10, 14 | 14 |
| 13 | 38.0, CH_2_ | 2.44, ddd (14.2, 9.6, 6.8), Ha |  | 37.5, CH_2_ | 2.22, ddd (14.0, 9.6, 6.8), Ha | 12, 13b, 14 | 9, 10, 14, 15 |  |
|  |  | 1.46, ddd (14.2, 8.2, 2.3), Hb |  |  | 1.38, ddd (14.0, 8.6, 2.6), Hb | 12, 13a, 14 | 9, 10, 14, 15 | 9, 12-OH, 17 |
| 14 | 47.0, CH | 2.13 |  | 46.8, CH | 1.92 | 9, 13a, 13b | 8, 9, 10, 12, 13, 15, 16, 17 | 7b, 8a, 12, 16b |
| 15 | 144.6, C |  |  | 145.4, C |  |  |  |  |
| 16 | 112.1, CH_2_ | 4.75, br s, Ha |  | 111.6, CH_2_ | 4.70, br s, Ha | 17 | 14, 15, 17 |  |
|  |  | 4.63, br s, Hb |  |  | 4.57, br s, Hb | 17 | 13, 14, 15, 17 | 8a, 14 |
| 17 | 18.7, CH_3_ | 1.68, br s |  | 18.4, CH_3_ | 1.64, br s | 16a, 16b | 14, 15, 16 | 9, 13b |
| 6-OH |  |  |  |  | 5.55, s |  | 1, 5, 6, 7 |  |
| 12-OH |  |  |  |  | 5.24, d (4.7) | 12 | 10, 12, 13 | 9, 11, 13b |

*^a^* Measured in CDCl_3_ (400 MHz for ^1^H and 100 MHz for ^13^C).

*^b^* Measured in DMSO-*d*_6_ (600 MHz for ^1^H and 150 MHz for ^13^C).

*^c^* Indiscernible signals from overlap or complex multiplicity are reported without designating multiplicity.

**3. General experimental procedures**

UV data were recorded using a JASCO V-550 UV/vis spectrometer (Jasco International Co. Ltd, Tokyo, Japan). IR data were recorded on a JASCO FT/IR-4600 plus spectrometer (Jasco International Co. Ltd, Tokyo, Japan). Optical rotations were measured on a JASCO P2000 digital polarimeter (Jasco International Co. Ltd, Tokyo, Japan). ESI-MS spectra were performed on a Bruker amazon SL mass spectrometer (Bruker Corporation, Boston, USA). HRESIMS spectra were obtained on a Waters Synapt G2 TOF mass spectrometer (Waters Corporation, Milford, USA). ECD spectra were recorded in MeOH using a JASCO J-810 spectrophotometer (Jasco International Co. Ltd, Tokyo, Japan) at room temperature. NMR spectra were acquired with Bruker AV 300, Bruker AV 400, and Bruker AV 600 (Bruker BioSpin Group, Faellanden, Switzerland) using the solvent signals (DMSO-*d*_6_: *δ*_H_ 2.50/*δ*_C_ 39.5; CDCl_3_: *δ*_H_ 7.26/*δ*_C_ 77.0; CD_3_OD: *δ*_H_ 3.30/*δ*_C_ 49.0) as internal standards. A analytical HPLC was performed on a Dionex HPLC system equipped with an Ultimate 3000 pump, an Ultimate 3000 diode array detector (DAD), an Ultimate 3000 Column Compartment, and an Ultimate 3000 autosampler (Thermo Fisher Scientific Inc., Sunnyvale, USA) using a Phenomenex Gemini C18 column (4.6 × 250 mm, 5 μm) (Phenomenex Inc., Los Angeles, USA). Preparative HPLC was carried out on a Shimadzu LC-6-AD Liquid Chromatography system with a SPD-20A Detector using a Phenomenex Gemini C18 column (10.0 × 250 mm, 5 μm) (Phenomenex Inc., Los Angeles, USA). Medium pressure liquid chromatography (MPLC) was performed with a dual pump gradient system, a UV preparative detector, and a Dr Flash II fraction collector system (Lisui E-Tech Co. Ltd, Shanghai, China). Column chromatography (CC) was carried out on silica gel (Haiyang Chemical Co. Ltd., Qingdao, China), and ODS (50 μm) (YMC Co. Ltd, Tokyo, Japan).

**4. Fungal material**

The strain numbered as J13-2-12Y was isolated from the leaves of *A. tatarinowii* collected from Guangxi Medicinal Botanical Garden, Guangxi Province, People's Republic of China. After washed with sterile water for 10 min, leaf sample was surface-sterilized by consecutive immersion for 2 min in 75% ethanol. The surface of surface-sterilized sample was dried with sterile paper towels and cut into pieces with 2 cm. After that, the pieces were placed in 90 mm Petri dish containing potato dextrose agar (PDA). Chloramphenicol (100 mg/L) was added to PDA in order to suppress bacterial growth. Petri dishes were sealed, incubated at 25°C, and examined periodically. When fungal colonies developed, they were transferred to new Petri dishes with PDA for purification. Vouched pure strains were transferred to PDA slants for molecular identification and chemical investigation.

The ribosomal internal transcribed spacer (ITS) and the 5.8S rRNA gene sequences (ITS1-5.8S-ITS2) of the strain have been deposited at GenBank as KY964333.The fungus was cultured on slants of potato dextrose agar (PDA) at 25 °C for 5 days. Agar plugs were used to inoculate four Erlenmeyer flasks (250 mL), each containing 100 mL of potato dextrose broth (PDB). Fermentation was carried out in 25 Erlenmeyer flasks (500 mL), each containing 70 g of rice. Distilled H_2_O (110 mL) was added to each flask, followed by autoclaving at 120 °C for 30 min. After cooling to room temperature, each flask was inoculated with 5.0 mL of the mycelia-containing seed culture and incubated at room temperature for 39 days.

**5. Alkaline hydrolysis of 1 and 2**

A sample of **1** (1 mg) was treated with 2 N KOH (200 μL), THF (200μL), and CH_3_OH (200 μL), and stirred at 25 °C for 4 h. After neutralizing with 10% HCOOH and extracting with EtOAc, the EtOAc layer was evaporated to dryness and dissolved in MeOH. Then the mixture was purified on analytical HPLC (Phenomenex Gemini C18 column (4.6 × 250 mm, 5 μm)) with MeOH-H_2_O (69:31, v/v) at flow rate of 1 mL/min to yield **1a** (0.4 mg), whose ^1^H NMR spectrum and ECD spectrum were identical with those of **7.**

**Figure S1.** The products prepared from **1** and **7** were compared by HPLC with MeOH-H_2_O (69:31, v/v) elution.

**Figure S2.** ^1^H NMR spectra of **1a** and **7** in CDCl_3_ at 300 MHz

**Figure S3.** The experimental ECD spectra of **1a** and **7**

A sample of **2** (1 mg) was treated with 2 N KOH (200 μL), THF (200μL), and CH_3_OH (200 μL), and stirred at 25 °C for 4 h. After neutralizing with 10% HCOOH and extracting with EtOAc, the EtOAc layer was evaporated to dryness and dissolved in MeOH. Then the mixture was purified by analytical HPLC (Phenomenex Gemini C18 column (4.6 × 250 mm, 5 μm)) with MeOH-H_2_O (67:33, v/v) at flow rate of 1 mL/min to yield **2a** (0.4 mg), whose ^1^H NMR spectrum and ECD spectrum were identical with those of **8.**

**Figure S4.** The products prepared from **2** and **8** were compared by HPLC with MeOH-H_2_O (67:33, v/v) elution.

**Figure S5.** ^1^H NMR spectra of **2a** and **8** in CD_3_OD at 400 MHz

**Figure S6.** The experimental ECD spectra of **2a** and **8**

**6. Antimicrobial assay**

The antimicrobial activities against *S. aureus* 209P (bacterium) and *C. albicans* FIM709 (fungus) were measured in sterile 96-well plates using the broth microdilution method^1,2^. Beef extract agar media for bacterium and sabouraud’s dextrose agar media for fungus were used, respectively. These two media were sterilized by high temperature. Final sample concentrations were prepared from serial dilutions and ranged from 128 to 0.25 μg/mL (128, 64, 32, 16, 8, 4, 2, 1, 0.5, and 0.25 μg/mL) in the growth medium, and the continuous 2-fold dilution method was used to evaluate the minimal inhibitory concentrations (MICs). The MICs were defined as the lowest concentration at which no microbial growth could be observed. Itraconazole and tobramycin were used as the positive controls for the antifungal and antibacteria assays, respectively.

References:

1. Groblacher, B., Maier, V., Kunert, O., Bucar, F. Putative mycobacterial efflux inhibitors from the seeds of *Aframomum melegueta*. *J. Nat. Prod.* **75**, 1393–1399 (2012).
2. Qin, X. J., *et al*. Antibacterial prenylbenzoic acid derivatives from *Anodendron formicinum*. *Fitoterapia* **92**, 238–243 (2014).

**7. The 1D and 2D NMR spectra of 1−3**

**The 1D and 2D NMR spectra of phyllomeroterpenoid A (1)**

**Figure S7.** ^1^H NMR spectrum of **1** in CDCl_3_ at 600 MHz


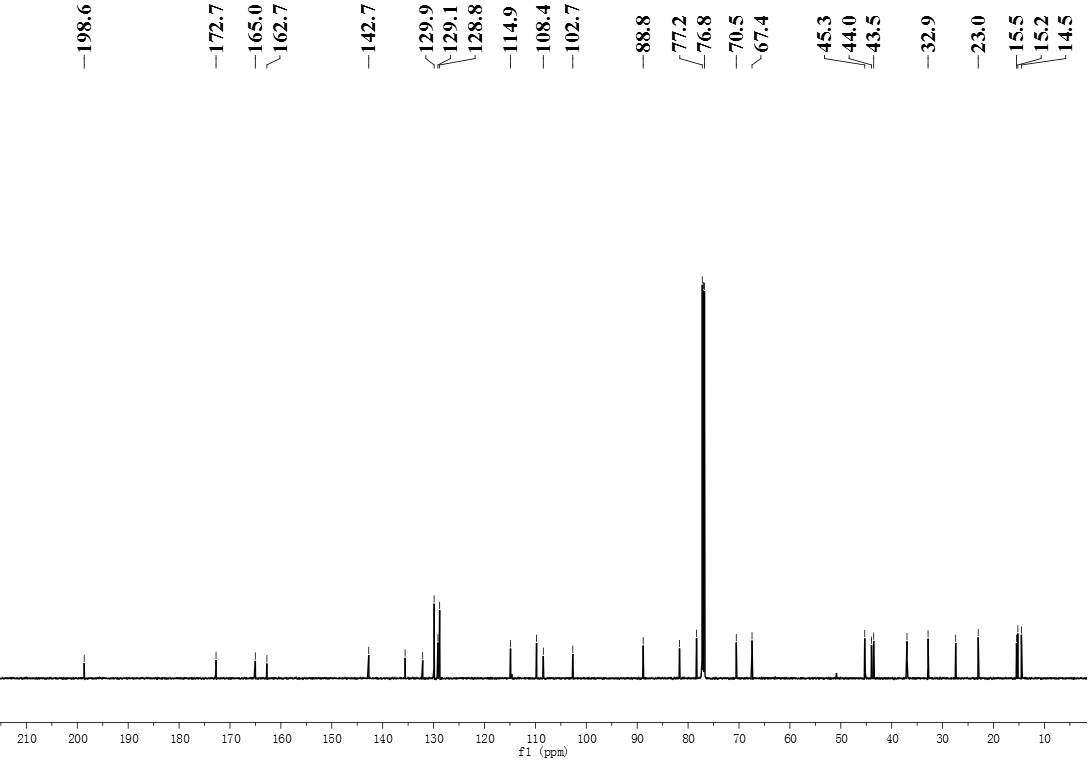


**Figure S8.** ^13^C NMR spectrum of **1** in CDCl_3_ at 150 MHz

**Figure S9.** HSQC spectrum of **1** in CDCl_3_

**Figure S10.** ^1^H-^1^H COSY spectrum of **1** in CDCl_3_

**Figure S11.** HMBC spectrum of **1** in CDCl_3_


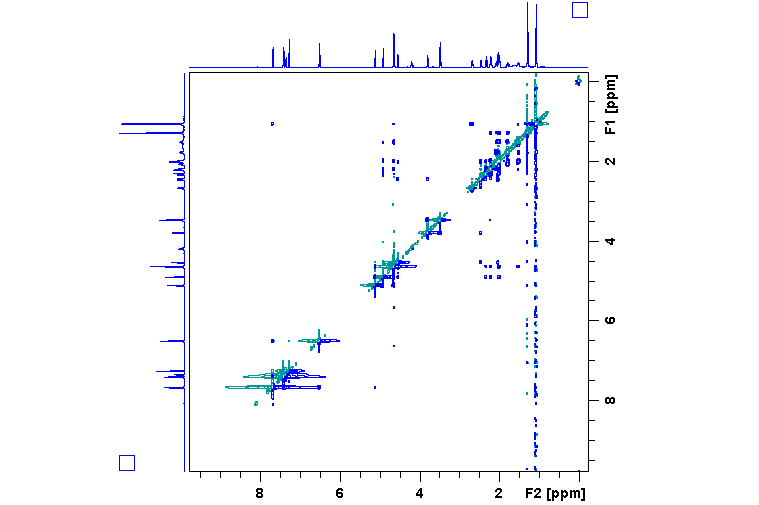


**Figure S12.** NOESY spectrum of **1** in CDCl_3_

**The 1D and 2D NMR spectra of phyllomeroterpenoid B (2)**

**Figure S13.** ^1^H NMR spectrum of **2** in CD_3_OD at 600 MHz

**Figure S14.** ^13^C NMR spectrum of **2** in CD_3_OD at 150 MHz

**Figure S15.** HSQC spectrum of **2** in CD_3_OD

**Figure S16.** ^1^H-^1^H COSY spectrum of **2** in CD_3_OD

**Figure S17.** HMBC spectrum of **2** in CD_3_OD


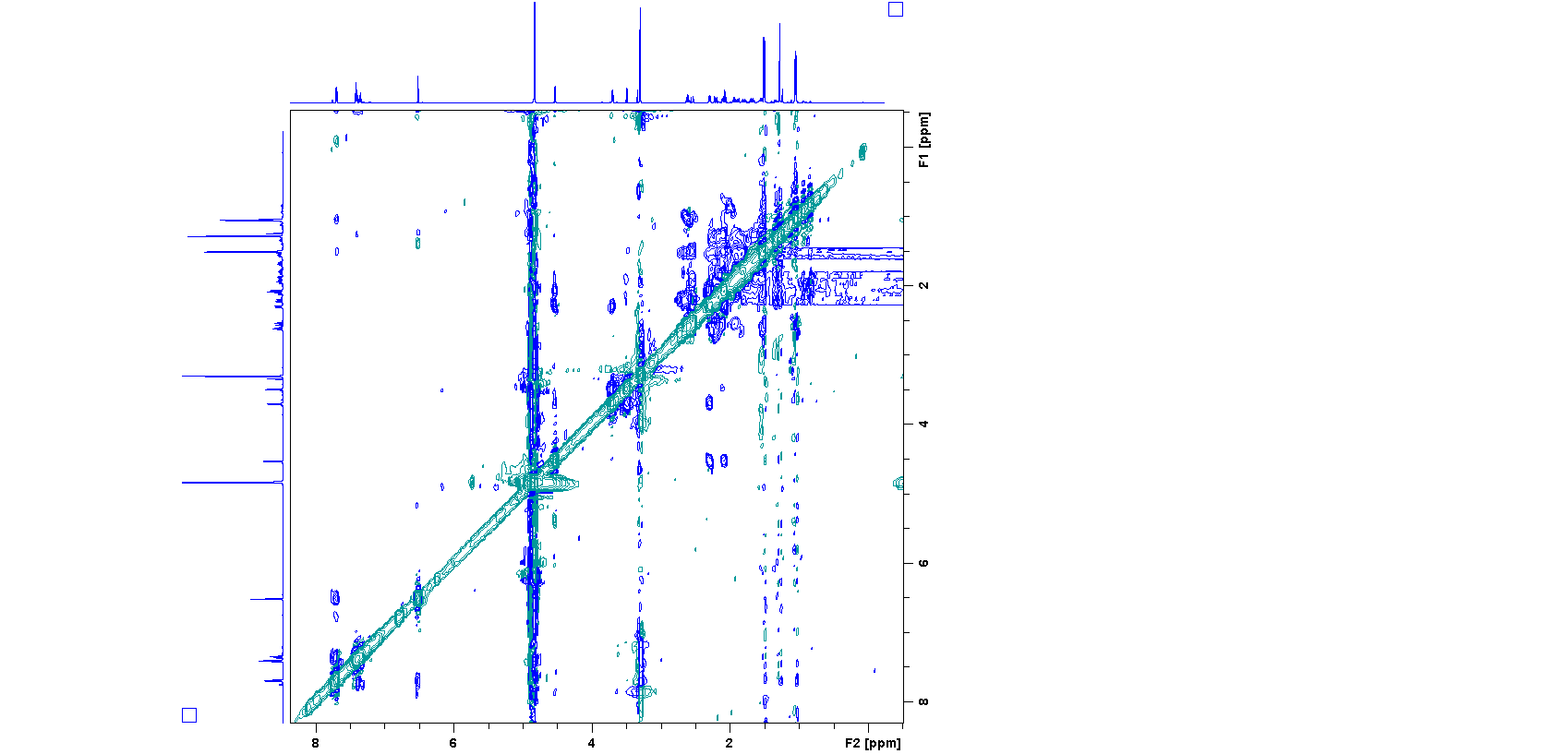


**Figure S18.** NOESY spectrum of **2** in CD_3_OD

**The 1D and 2D NMR spectra of phyllomeroterpenoid C (3)**

**Figure S19.** ^1^H NMR spectrum of **3** in CDCl_3_ at 600 MHz


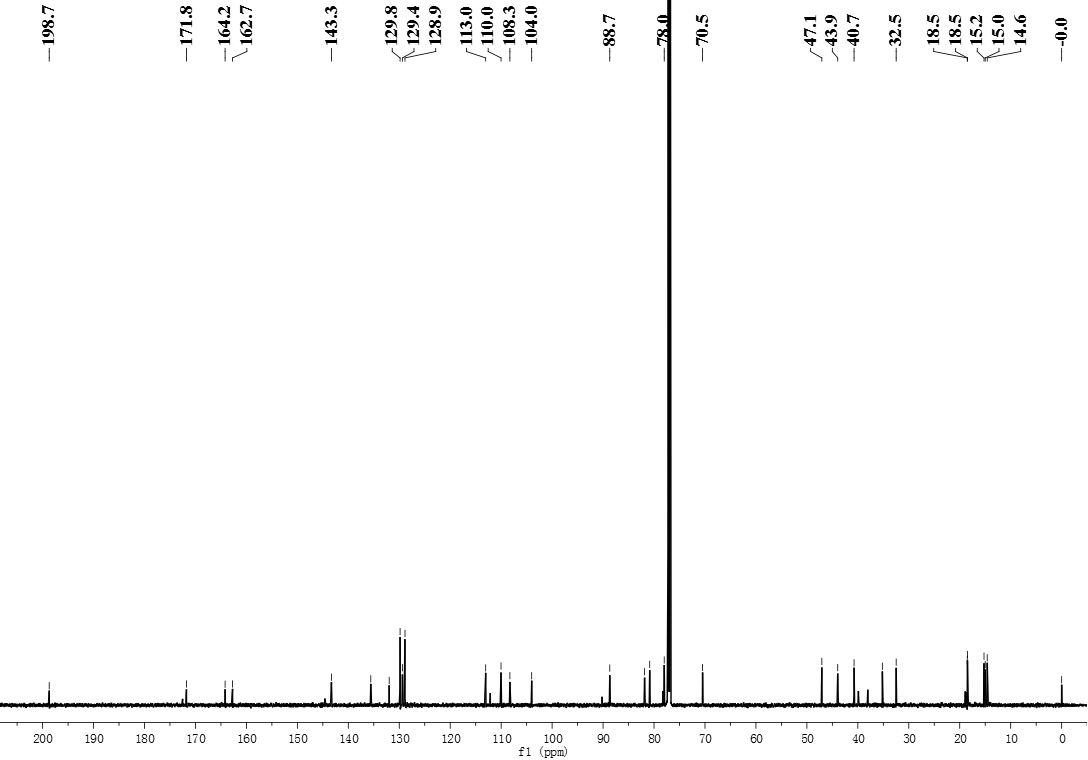


**Figure S20.** ^13^C NMR spectrum of **3** in CDCl_3_ at 150 MHz

**Figure S21.** HSQC spectrum of **3** in CDCl_3_

**Figure S22.** ^1^H-^1^H COSY spectrum of **3** in CDCl_3_

**Figure S23.** HMBC spectrum of **3** in CDCl_3_


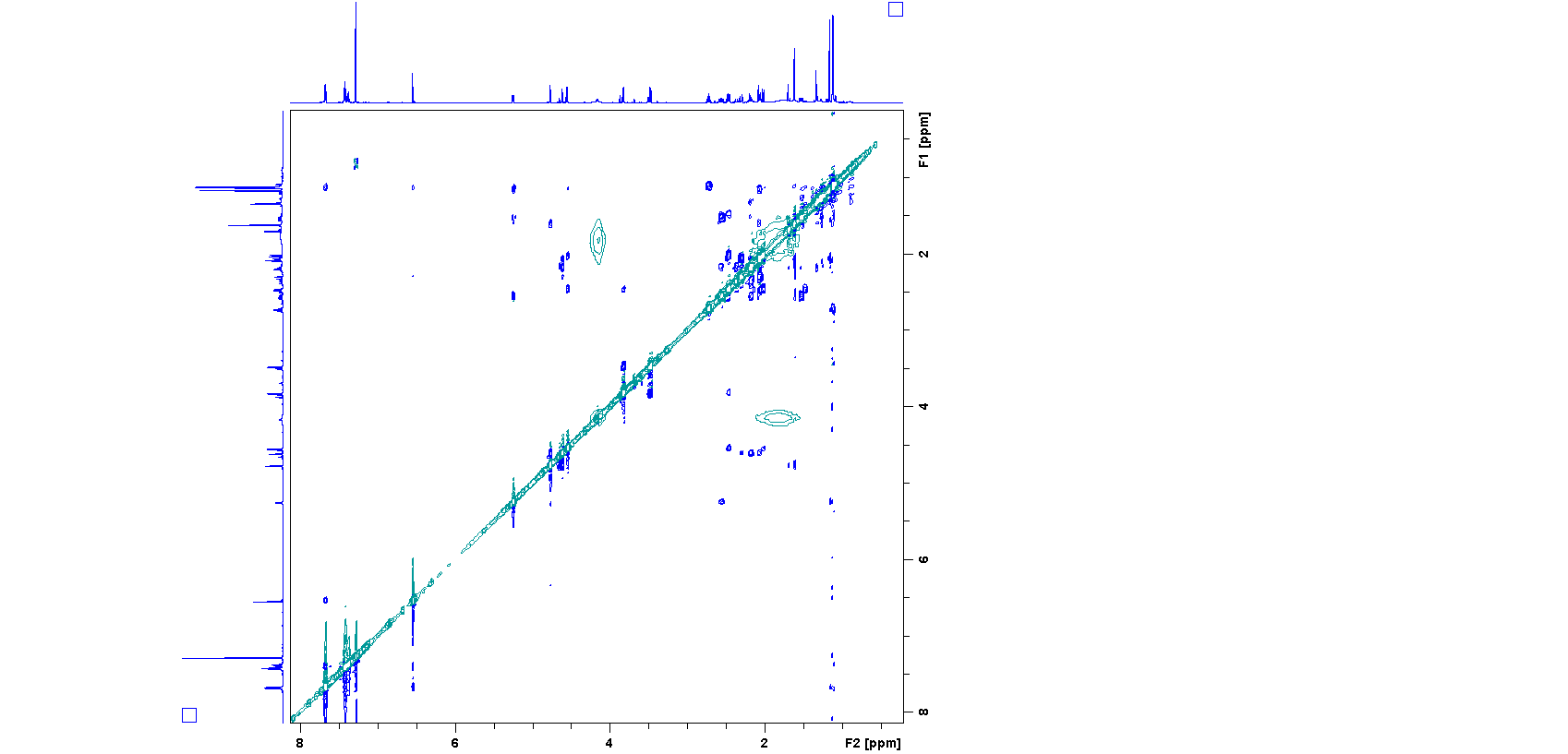


**Figure S24.** NOESY spectrum of **3** in CDCl_3_

**8. The 1D and 2D NMR spectra of 4, 8 and 9**

**The 1D and 2D NMR spectra of guignardianone C (4)**


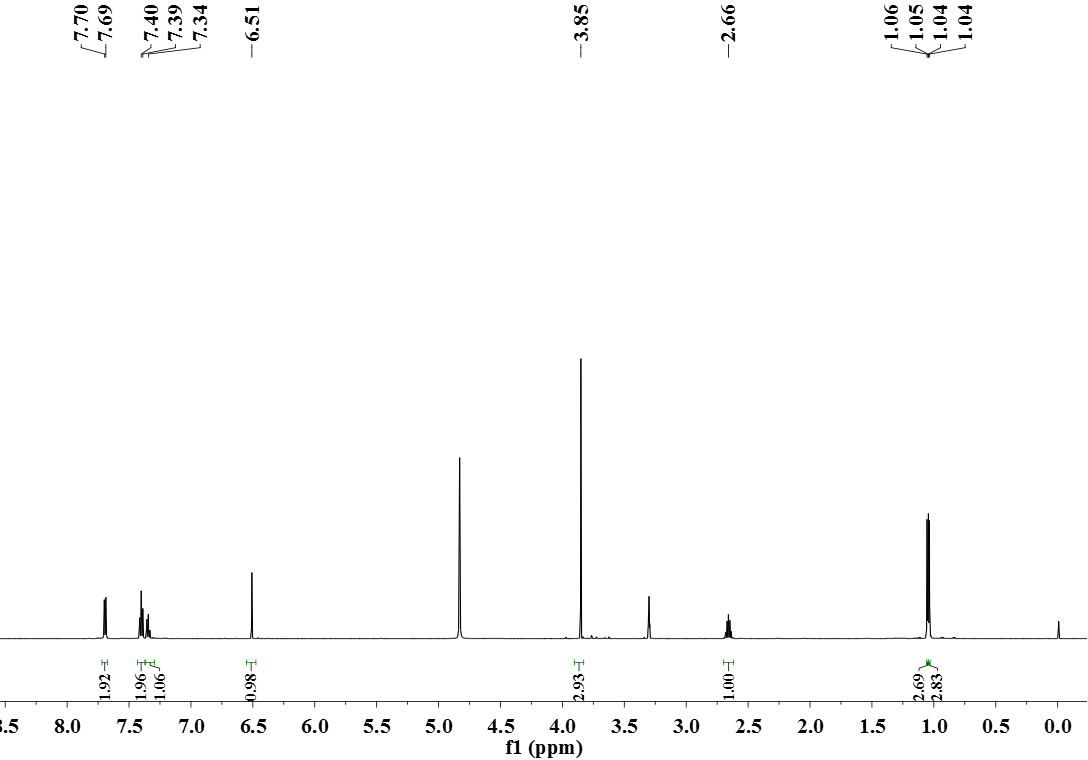


**Figure S25.** ^1^H NMR spectrum of **4** in CD_3_OD at 600 MHz


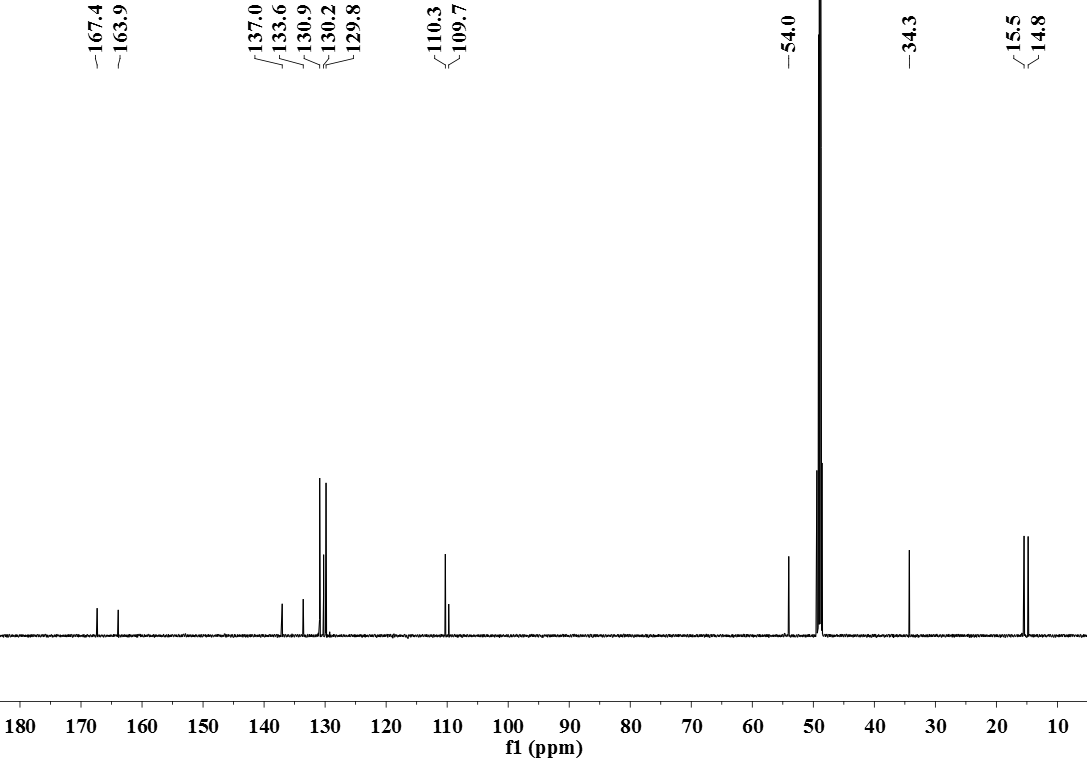


**Figure S26.** ^13^C NMR spectrum of **4** in CD_3_OD at 150 MHz


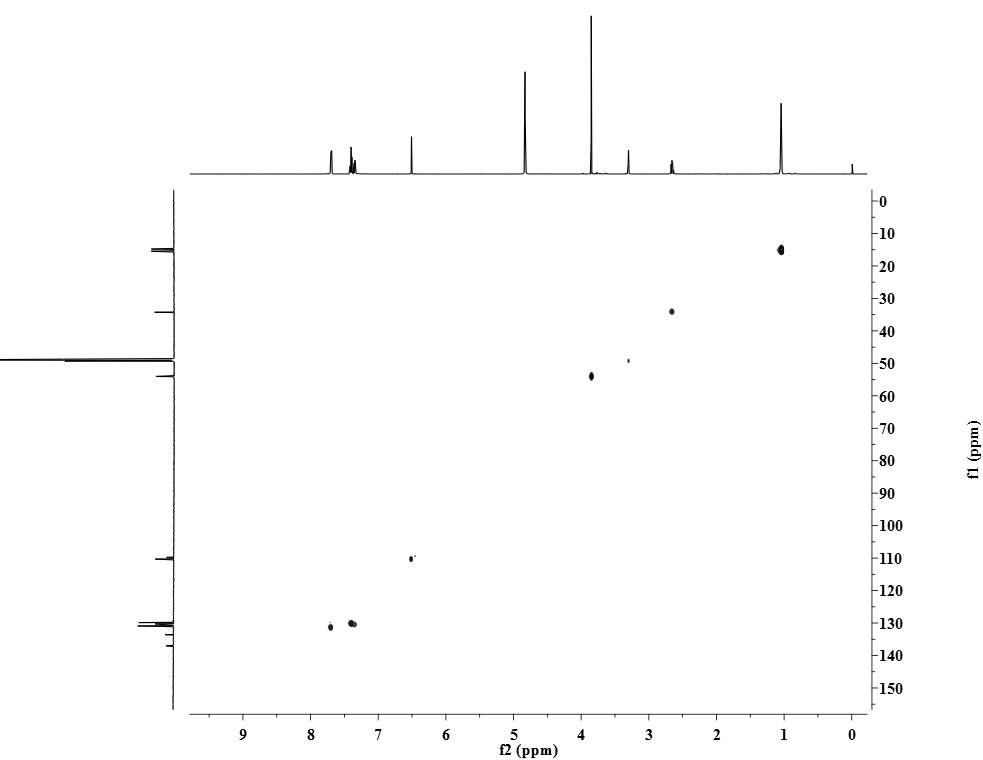


**Figure S27.** HSQC spectrum of **4** in CD_3_OD


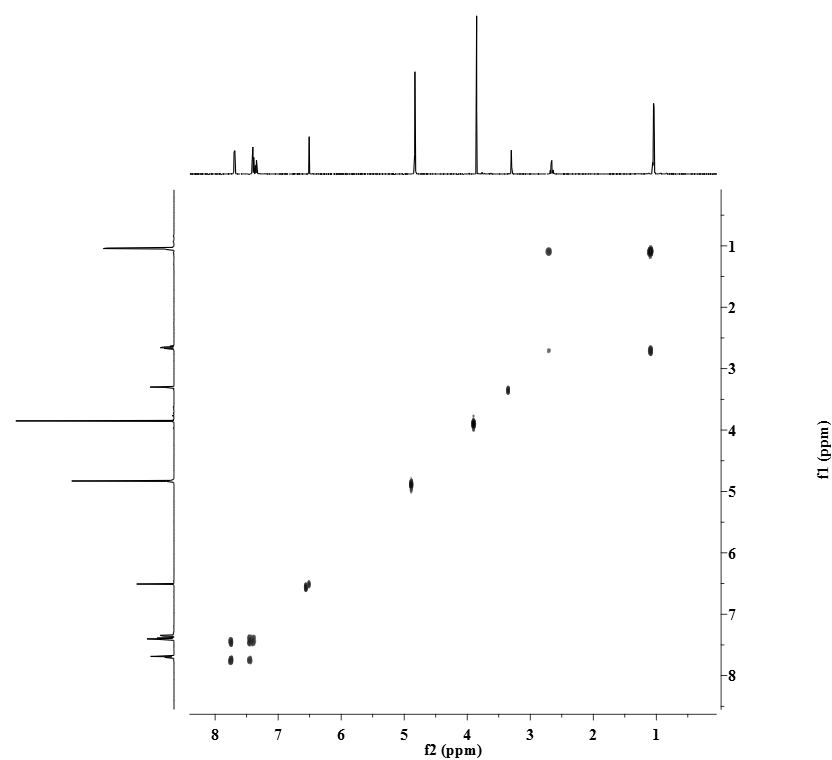


**Figure S28.** ^1^H-^1^H COSY spectrum of **4** in CD_3_OD


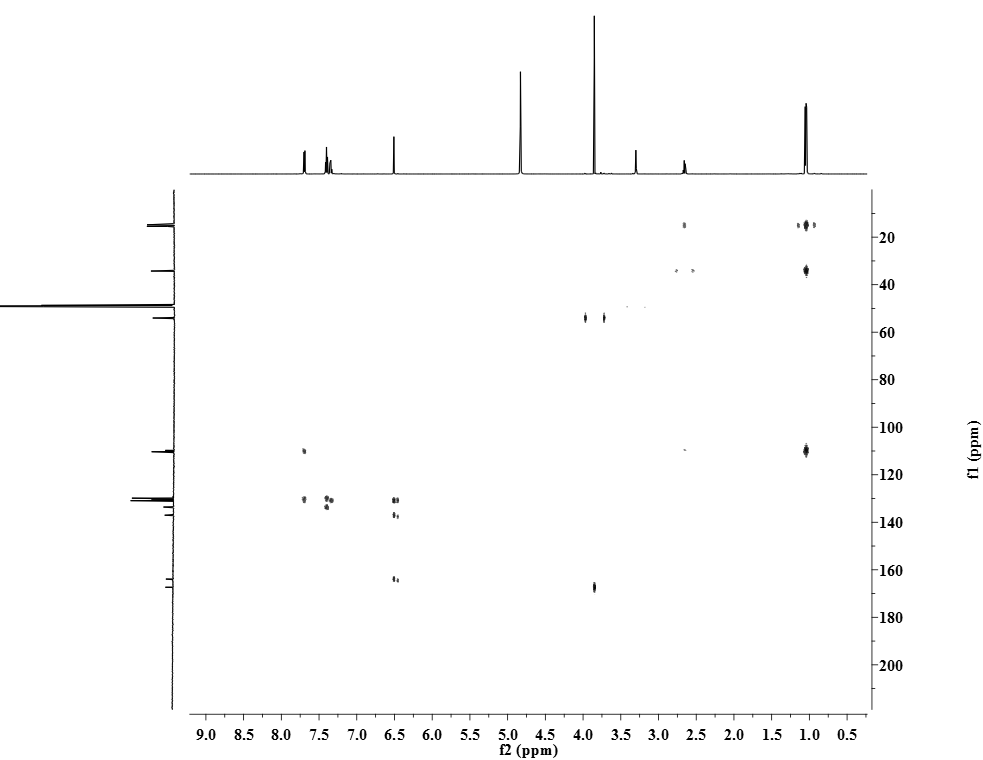


**Figure S29.** HMBC spectrum of **4** in CD_3_OD

**The 1D and 2D NMR spectra of guignardone B (8)**


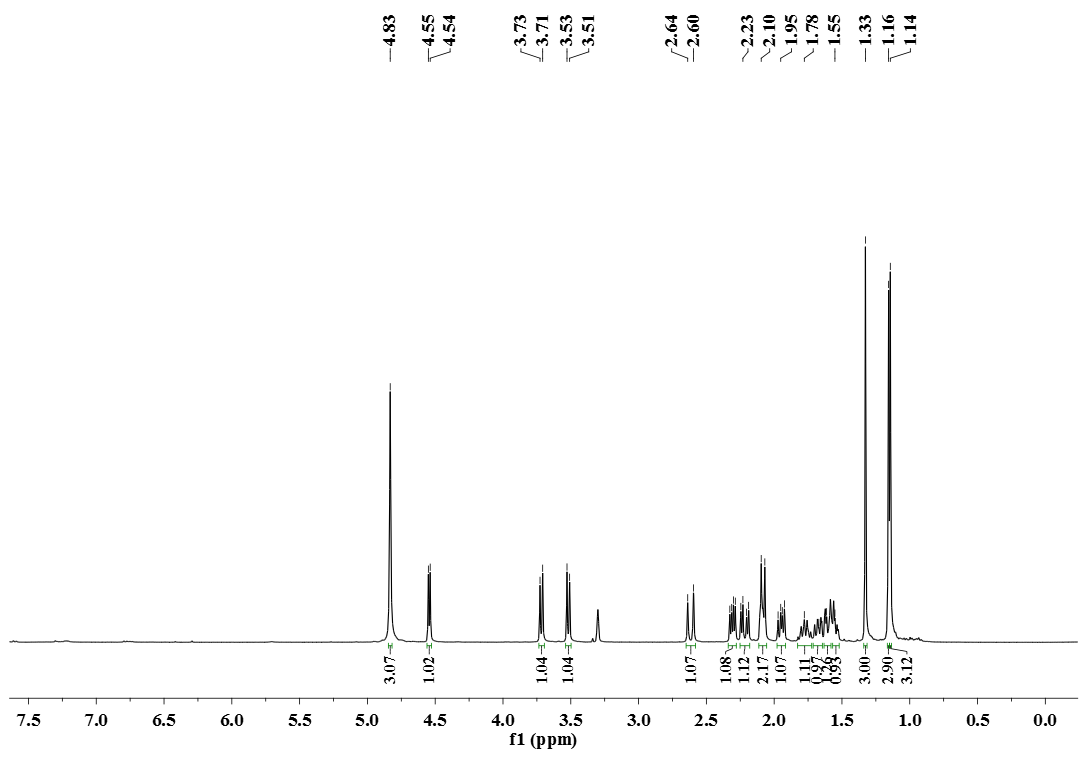


**Figure S30.** ^1^H NMR spectrum of **8** in CD_3_OD at 400 MHz


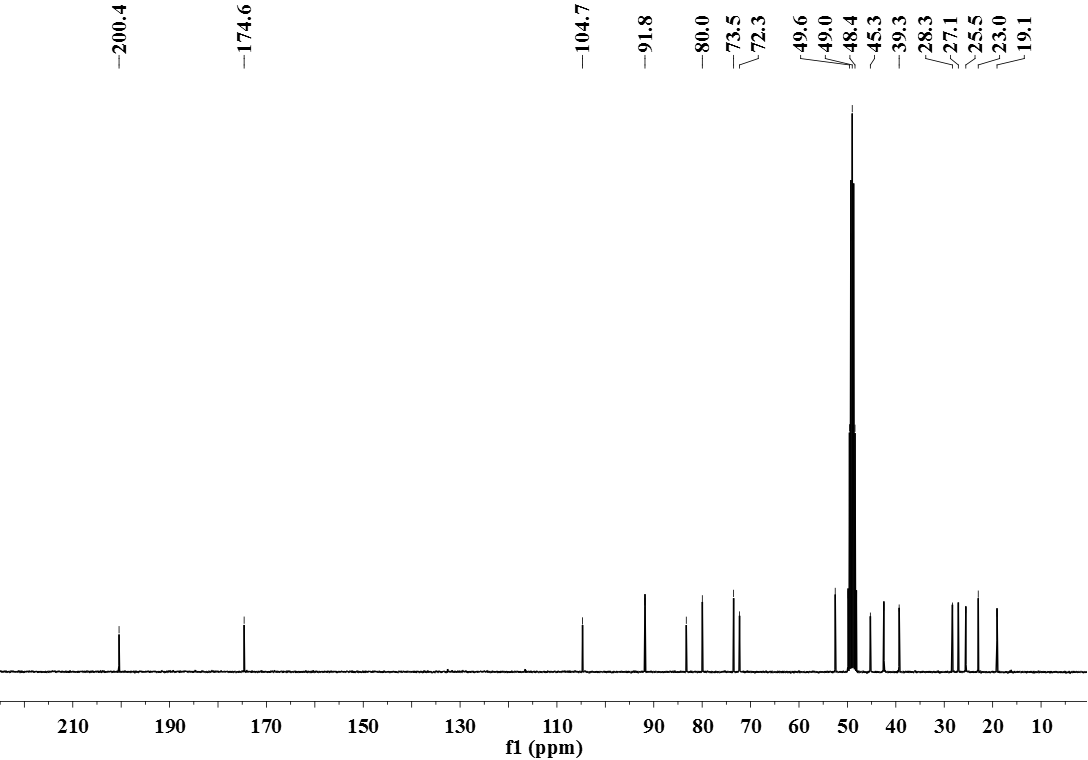


**Figure S31.** ^13^C NMR spectrum of **8** in CD_3_OD at 100 MHz


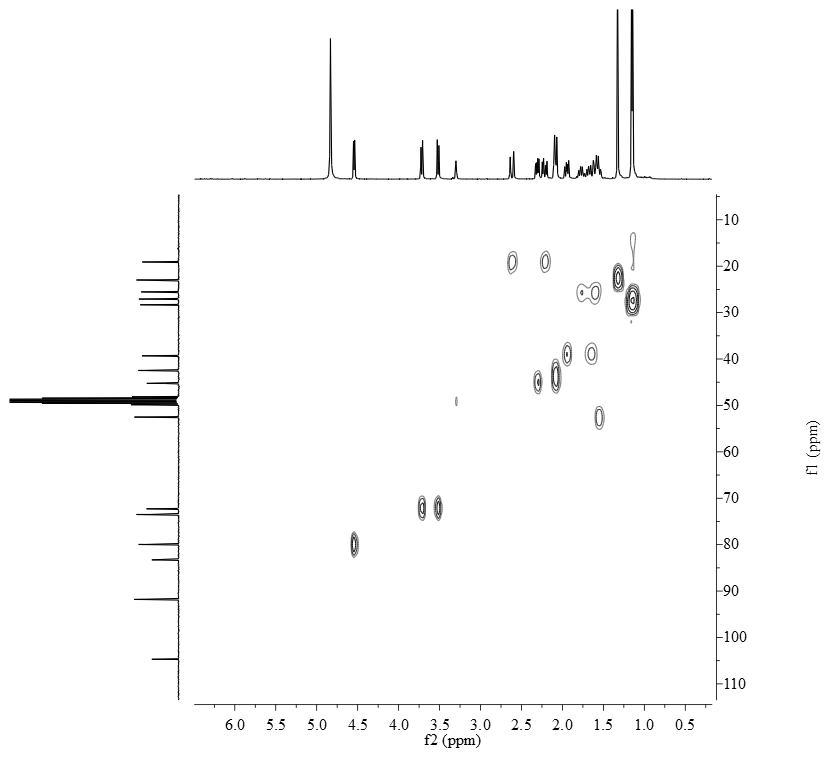


**Figure S32.** HSQC spectrum of **8** in CD_3_OD


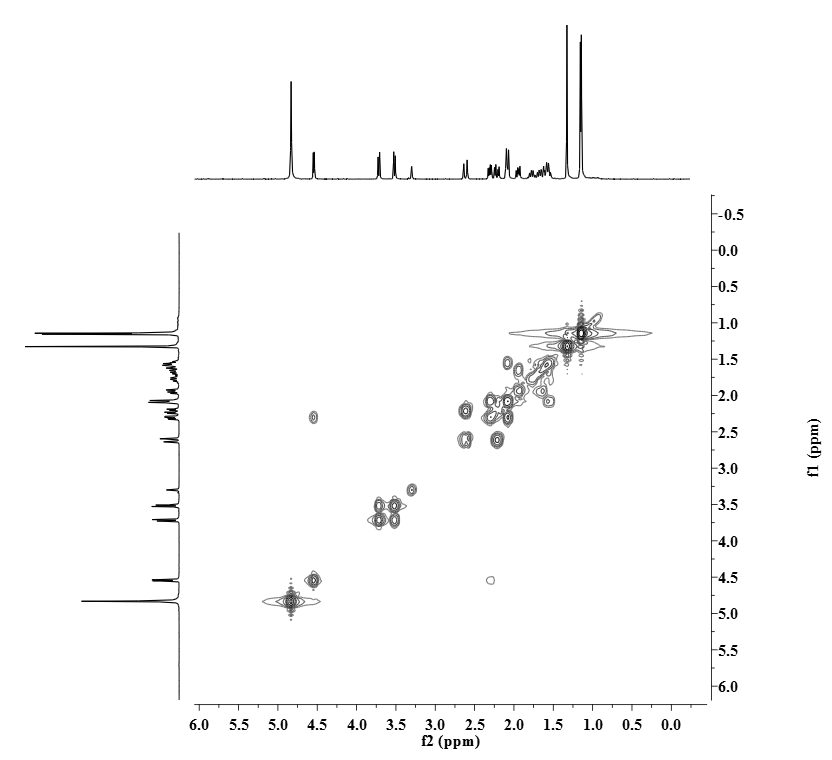


**Figure S33.** ^1^H-^1^H COSY spectrum of **8** in CD_3_OD


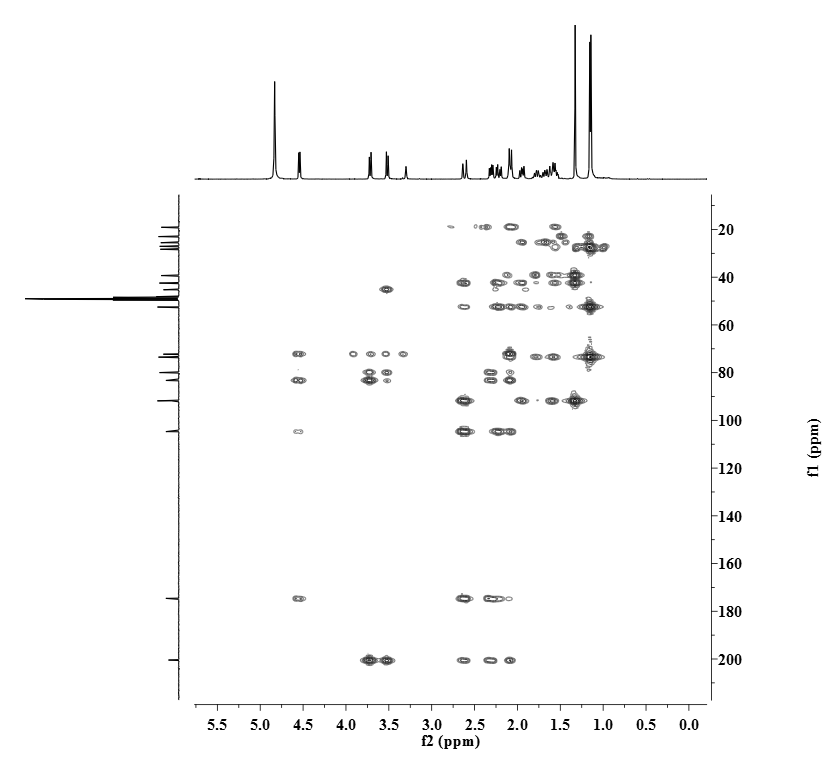


**Figure S34.** HMBC spectrum of **8** in CD_3_OD

**The 1D and 2D NMR spectra of 12-hydroxylated guignardone A (9)**


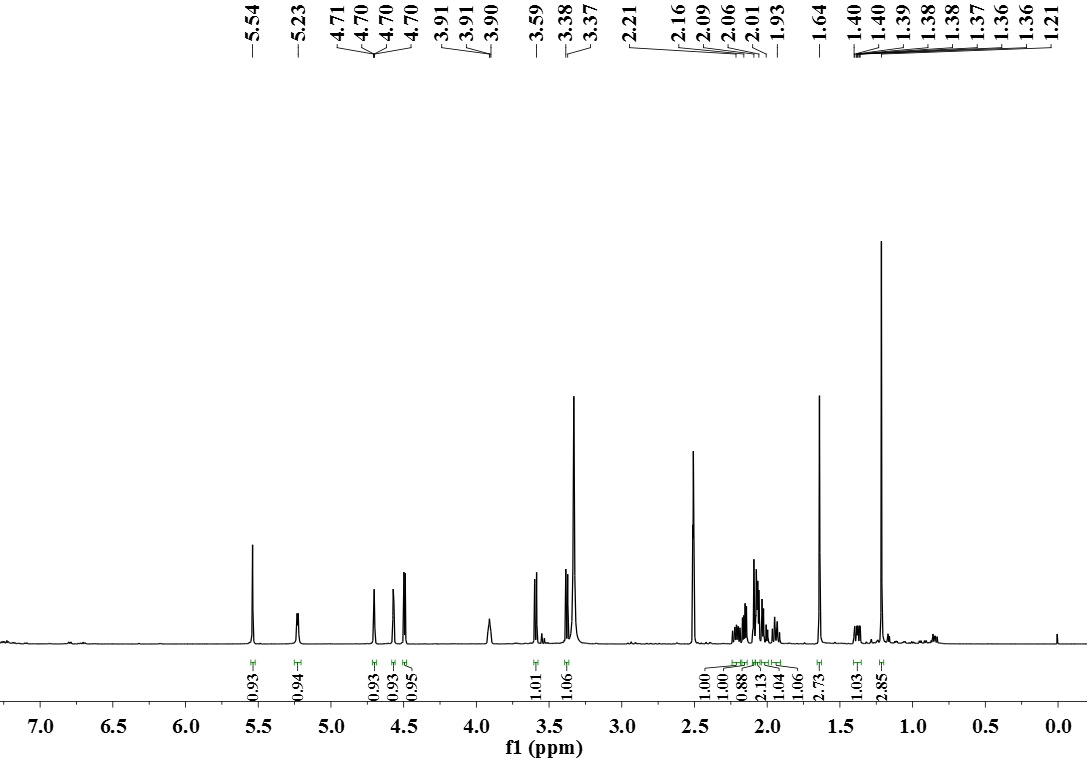


**Figure S35.** ^1^H NMR spectrum of **9** in DMSO-*d*_6_ at 600 MHz


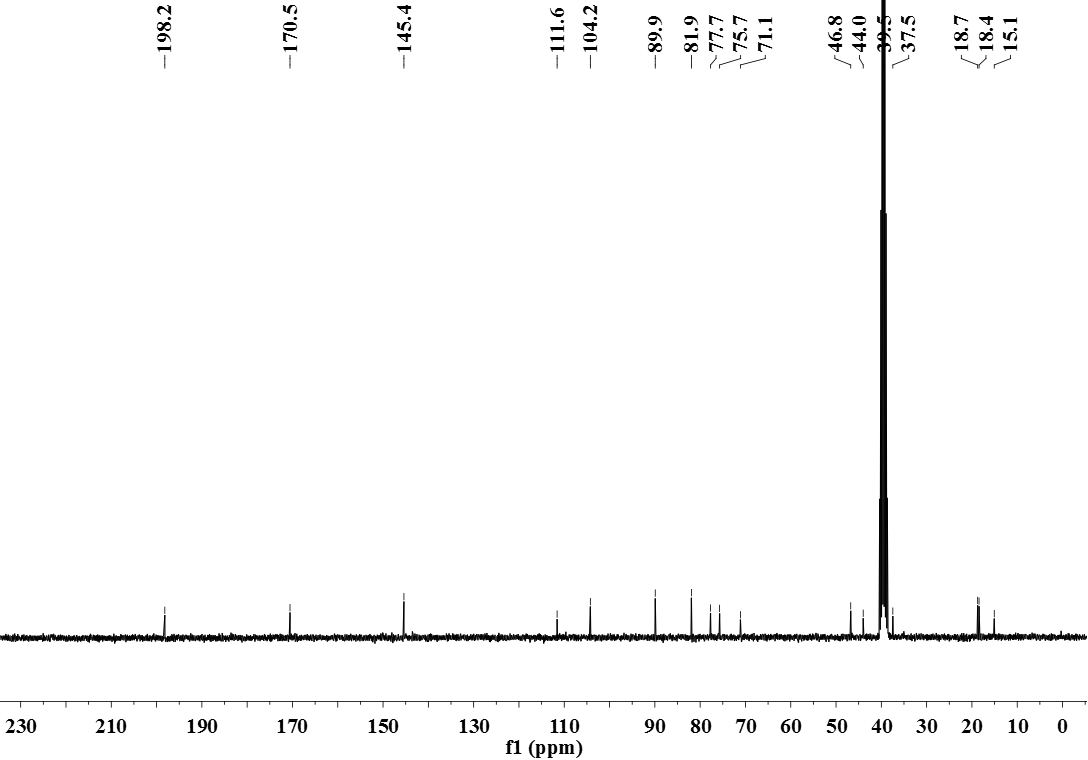


**Figure S36.** ^13^C NMR spectrum of **9** in DMSO-*d*_6_ at 150 MHz


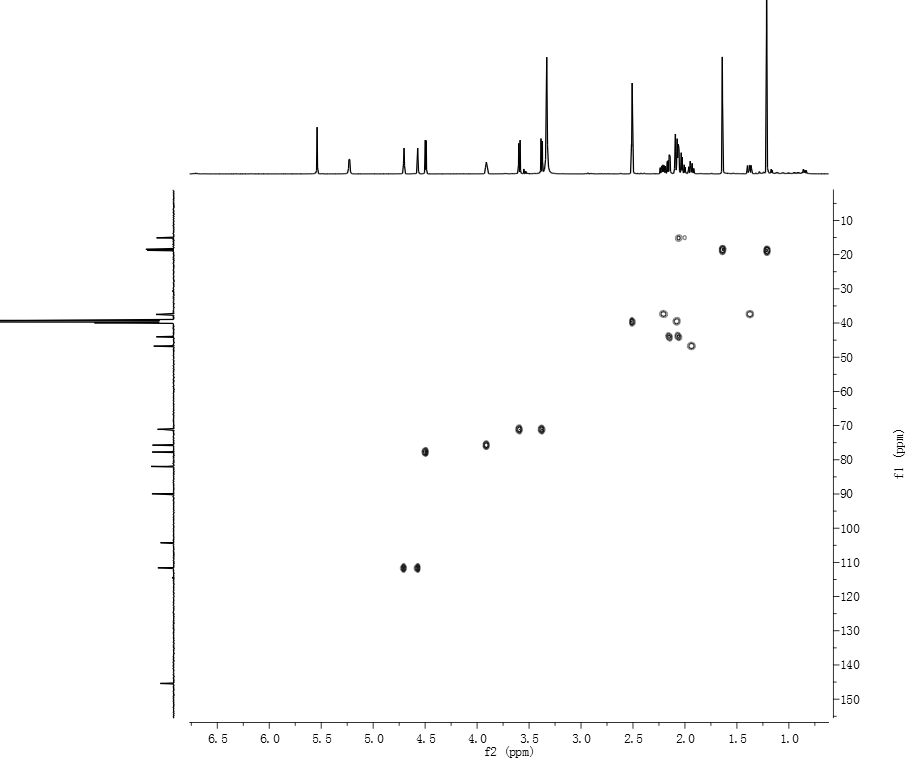


**Figure S37.** HSQC spectrum of **9** in DMSO-*d*_6_
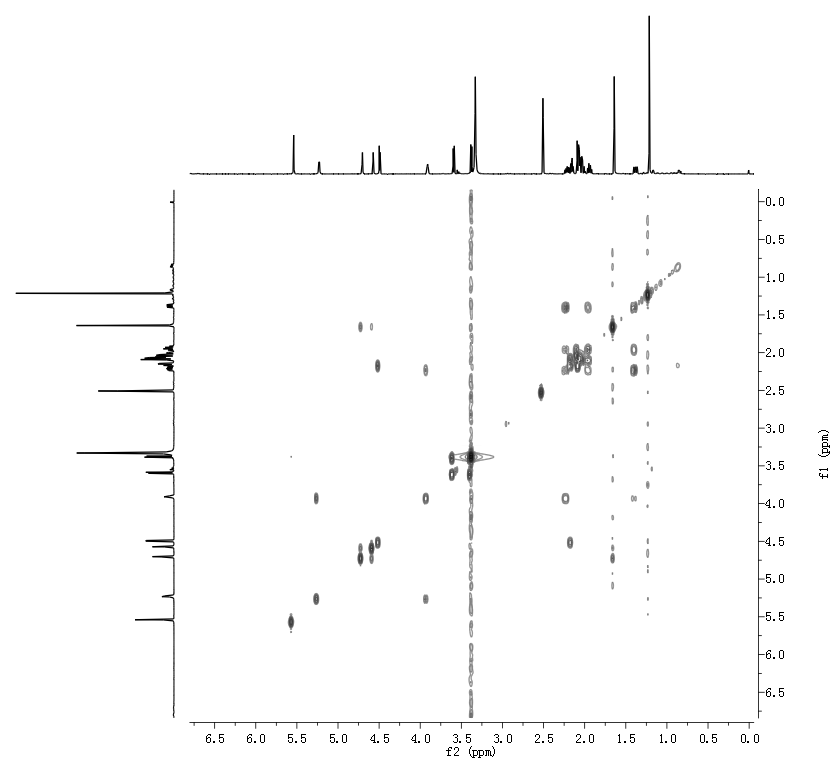


**Figure S38.** ^1^H-^1^H COSY spectrum of **9** in DMSO-*d*_6_
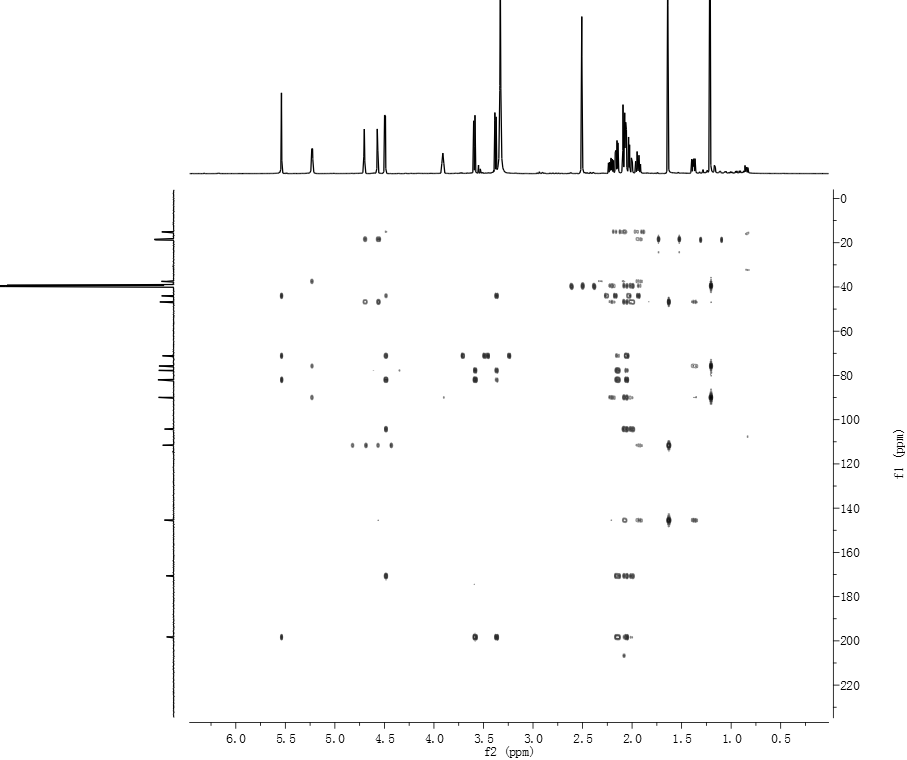


**Figure S39.** HMBC spectrum of **9** in DMSO-*d*_6_


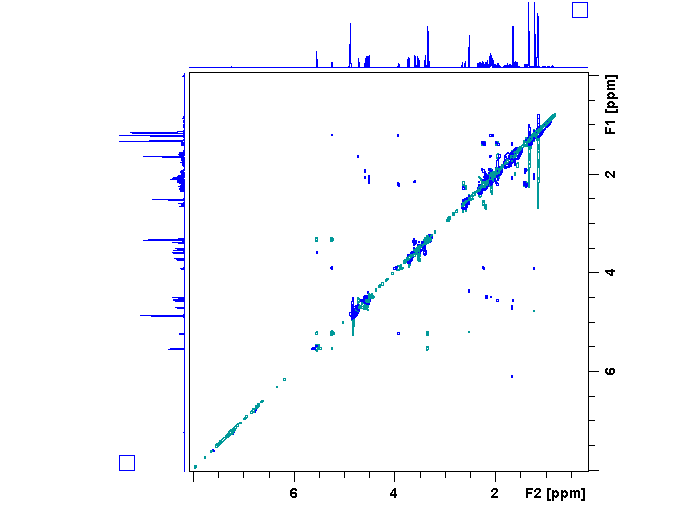


**Figure S40.** ROESY spectrum of **9** in DMSO-*d*_6_
